# Efficacy and Safety of Traditional Chinese Medicine in Coronavirus Disease 2019 (COVID-19): A Systematic Review and Meta-Analysis

**DOI:** 10.3389/fphar.2021.609213

**Published:** 2021-08-06

**Authors:** Heping Wang, Bowen Xu, Ying Zhang, Yuanyuan Duan, Ruike Gao, Haoqiang He, Xiuyang Li, Jie Li

**Affiliations:** ^1^ Department of Oncology, Guang’anmen Hospital, China Academy of Chinese Medical Sciences, Beijing, China; ^2^ Graduate School, China Academy of Chinese Medical Sciences, Beijing, China; ^3^ Graduate School, Beijing University of Chinese Medicine, Beijing, China; ^4^ Department of Respiratory, Guang’anmen Hospital, China Academy of Chinese Medical Sciences, Beijing, China; ^5^ Department of Cardiology, Guang’anmen Hospital, China Academy of Chinese Medical Sciences, Beijing, China; ^6^ Department of Medical Affairs, Guang’anmen Hospital, China Academy of Chinese Medical Sciences, Beijing, China

**Keywords:** traditional Chinese medicine, COVID-19, randomized controlled trial, systematic review, meta-analysis, SARS-CoV-2

## Abstract

**Introduction:** Until now, there is no clinically approved specific medicine to treat COVID-19. Prior systematic reviews (SRs) have shown that traditional Chinese medicine (TCM) reduces the number of patients with severe disease and time to fever clearance, promotes clinical effectiveness, and improves chest images and the negativity rate of severe acute respiratory syndrome coronavirus 2 (SARS-CoV-2) nucleic acid test. Few SRs arrived at a definitive conclusion, and more randomized controlled trials (RCTs) were published. We conducted this study to summarize the latest evidence of TCM in COVID-19.

**Methods:** Eight online databases were searched from December 2019 to July 2020, updated to March 2021. Only RCTs evaluating the clinical efficacy and safety of TCM in the treatment of COVID-19 were included. Primary outcomes were clinical cure and the negativity of the SARS-CoV-2 nucleic acid test. Secondary outcomes included clinical deterioration, ARDS, mechanical ventilation, death, time to fever clearance, duration of hospitalization, and chest imaging improvement. Safety outcomes included adverse events and serious adverse events during treatment. Two reviewers selected the included articles, assessed the risk of bias, and extracted data independently and in duplicate.

**Results:** A total of 25 RCTs involving 2222 participants were selected in the systematic review, and seven RCTs were included in the meta-analysis. The results showed that TCM plus routine treatment was significantly better than routine treatment alone in clinical cure (risk ratio [RR] = 1.20, 95% confidence interval (CI) [1.04, 1.38], *P* = 0.01) and chest image improvement (RR = 1.22, 95% CI [1.07, 1.39], *P* = 0.01) and could reduce clinical deterioration (RR = 0.39, 95% CI [0.18, 0.86], *P* = 0.02), ARDS (RR = 0.28, 95% CI [0.11, 0.69], *P* = 0.01), mechanical ventilation (RR = 0.30, 95% CI [0.12, 0.77], *P* = 0.01), or death rate (RR = 0.28, 95% CI [0.09, 0.84], *P* = 0.02). No significant difference between TCM and routine treatment in the negativity of SARS-CoV-2 nucleic acid test (RR = 1.08, 95% CI [0.94, 1.23], *P* = 0.29) was observed. Finally, there was no overall significant difference in the incidence of adverse events between the two groups. The summary of evidence showed moderate confidence of a benefit of 11.8% in clinical cure and 14.0% in chest image improvement and a reduction of 5.9% in clinical deterioration, 25.4% in ARDS, 18.3% in mechanical ventilation, and 4.5% in death with TCM plus routine treatment compared to routine treatment alone in patients with COVID-19. A low confidence of a benefit of 5.4% in the negativity of SARS-CoV-2 nucleic acid test was also observed.

**Conclusions:** Synethized evidence of 21 outcomes in 8 RCTs showed moderate certainty that TCM treatment plus routine treatment may promote a clinical cure and chest image improvement compared to routine treatment alone while reducing clinical deterioration, development of ARDS, use of mechanical ventilation, and death in patients with COVID-19. TCM treatment plus routine treatment may not promote the negativity of the SARS-CoV-2 nucleic acid test compared to routine treatment alone. TCM treatment was found to be safe for patients with COVID-19.

## Introduction

Coronavirus disease 2019 (COVID-19) is a new acute respiratory infectious disease, and the global epidemic is still spreading since the outbreak in December 2019, becoming a major global public health event. Through active prevention, control, and treatment, the epidemic situation in China has been basically controlled, with only minor local outbreaks and a few imported cases from abroad in individual areas, whereas the epidemic situation in other countries remains difficult. There are still no effective clinical therapeutic drugs that can cure the disease.

Traditional Chinese medicine (TCM) has been used in the whole process of the novel coronavirus disease treatment in China, and the “Diagnosis and Treatment Protocol for Novel Coronavirus Pneumonia Trial Version 3” clearly stated that 91.5% (or 74,187) of COVID-19 patients were treated with Chinese herbal medicine (CHM) (National Health Commission of the People’s Republic of China, 2020a; The State Council Information Office of the People’s Republic of China, 2020). A large number of clinical studies have shown that early intervention with CHM and integrated traditional Chinese and western medicine can reduce clinical symptoms, shorten the course of the disease, prevent severe forms of the disease, improve the cure rate, and reduce mortality (Gao et al., 2020; Ren et al., 2020; Yang Y. et al., 2020).

Although more than 20 systematic reviews (SRs) were conducted to evaluate the clinical efficacy of TCM on the treatment of COVID-19 (Ang et al., 2020; Cai et al., 2020; Fan et al., 2020; Jin L. et al., 2020; Liang et al., 2020; Liu et al., 2020; Luo et al., 2020; Pang et al., 2020; Qi et al., 2020; Sun C.-Y. et al., 2020; Wang S. X. et al., 2020; Wu et al., 2020; Xiong X. et al., 2020; Zeng et al., 2020; Zhang H. Y. et al., 2020; Zhang W. B. et al., 2020; Zhou Z. et al., 2020; Gao et al., 2021; Liu M. et al., 2021; Ouyang et al., 2021; Zhou et al., 2021), most of them did not assess the quality of evidence and did not arrived at a definite conclusion (Ang et al., 2020; Cai et al., 2020; Fan et al., 2020; Jin L. et al., 2020; Liu et al., 2020; Qi et al., 2020; Sun C.-Y. et al., 2020; Wang S. X. et al., 2020; Xiong X. et al., 2020; Zeng et al., 2020; Zhang H. Y. et al., 2020; Zhang W. B. et al., 2020; Gao et al., 2021; Liu M. et al., 2021; Ouyang et al., 2021; Zhou et al., 2021). What is more, in 12 previously published SRs (Ang et al., 2020; Jin L. et al., 2020; Luo et al., 2020; Pang et al., 2020; Qi et al., 2020; Sun C.-Y. et al., 2020; Wang S. X. et al., 2020; Xiong X. et al., 2020; Zeng et al., 2020; Zhang H. Y. et al., 2020; Zhang W. B. et al., 2020; Liu M. et al., 2021), the authors did not evaluate the eligibility and quality of the included trials, retrospective observational studies were mistakenly regarded as randomized controlled trials (RCTs), and these SRs included synthesized data of observational studies with RCTs in the meta-analysis. One prior SR included a trial of suspected cases of COVID-19 (Fan et al., 2020). In addition, RCTs of TCM published recently were not included in previous SRs. For example, a rigorous double-blinded RCT was not included in all the previously published SRs; this study demonstrated that Xuebijing injection might suppress the cytokine storm in severe cases of COVID-19 patients (Luo et al., 2021). The current study was guided by the following questions. Can TCM treatment 1) promote clinical cure, 2) accelerate the clearance of SARS-CoV-2, and/or 3) prevent unfavorable clinical outcomes (e.g., health deterioration, ARDS, use of mechanical ventilation, or death) when integrated with western medicine? 4) How confident are we of the answers obtained? In addition, 5) is TCM treatment safe for COVID-19 patients?

The objective of this study was to perform a SR and meta-analysis of low risk of bias RCTs to evaluate the available evidence on clinical efficacy and safety of TCM in the treatment of COVID-19.

## Materials and Methods

This SR was guided by the Preferred Reporting Items for Systematic Reviews and Meta-Analyses (PRISMA) statement and checklist (Moher et al., 2009) (Additional File 1). This study was registered on PROSPERO (No. CRD42020171564). We updated the PROSPERO record on April 21, 2020. This study also followed an unpublished written protocol.

### Eligibility Criteria

#### Type of Studies

This SR included RCTs and excluded observational and animal studies because evidence obtained from RCTs is more convincing (Balshem et al., 2011). The meta-analysis only included outcomes assessed as low risk of bias.

#### Types of Participants

This SR included participants diagnosed with COVID-19 through etiological or serological tests. Mild, ordinary, severe, and critical cases were included, and clinical classifications followed the Diagnosis and Treatment Protocol of COVID-19 (National Health Commission of the People’s Republic of China, 2020b).

#### Types of Intervention and Control

Randomized studies of Chinese medicine interventions as the sole treatment or combined with other treatments were included in this study. Chinese medicine interventions include Chinese medicine formulas (e.g., Qingfei Paidu decoction, Huashi Baidu formula, and Xuanfei Baidu formula), Chinese patented medicine (e.g., Jinhua Qinggan granule and Lianhua Qingwen capsule), and Chinese medicine injections (e.g., Xuebijing and Xiyanping injections). Non-pharmacological studies were excluded. Placebo, standard medication treatment, and usual care were included as control groups. Usual care recommended by NHS’s protocol includes rest in bed, support therapy, ensuring sufficient caloric intake, monitoring water and electrolyte balance, monitoring vital signs, and oxygen saturation; standard medication treatment recommended by NHS’s protocol includes antiviral treatment (alpha interferon, lopinavir/ritonavir, ribavirin, chloroquine phosphate, and Arbidol) and antibiotic drug treatment (National Health Commission of the People’s Republic of China, 2020b).

#### Types of Outcomes

Randomized studies reporting outcomes related to clinical efficacy and safety of TCM in COVID-19 treatment were included in this study.

### Search Strategy

We searched PubMed, EMBASE, CENTRAL, Web of Science, the Chinese Biomedical Literature Database (CBM), the China National Knowledge Infrastructure (CNKI), the Wanfang database, and the Chinese Scientific Journals Database (VIP database). Initial database searches were performed from December 2019 to July 2020 and were updated in March 2021. The language was restricted to English and Chinese. We also searched the Chinese Clinical Trial Registry (ChiCTR) and ClinicalTrials.gov to identify ongoing and completed trials. RCTs included in previously published SRs and meta-analysis were additional records in our comprehensive search.

The search strategy was a combination of controlled vocabulary (MeSH terms and Emtree terms) and free-text terms. The search strategy for PubMed is shown in Additional File 2. Modifications to the search strategy were used with other databases.

### Screening and Selection

Search results were imported to EndNote X8. Two authors reviewed the titles and abstracts in the database search results after duplicates were removed. The full text was then reviewed and assessed for its eligibility. Screening and selection were independently processed in duplicate by the two reviewers. RCTs that met the inclusion criteria were included. The process is summarized using a PRISMA flow diagram.

### Data Extraction

The following data were extracted from the included studies: 1) identification information (first author and year of publication); 2) general information (study setting, sample size, and duration); 3) participants (clinical classification of COVID-19, age, and sex); 4) intervention details (type of Chinese medicine intervention, routes of delivery, name of Chinese patented medicine or formula, dose, frequency, and duration); 5) comparison details (name, dose, frequency, and duration of treatment); 6) outcomes details. Authors of the trials were contacted for any missing or incomplete data. The composition of formulation and patented drugs will be reported in botanical scientific names, not the Latin drug names used in pharmacopeia to avoid confusion (Rivera et al., 2014).

### Outcome Justification and Prioritization

Because the specific outcomes reported in the included studies were somewhat inconsistent with our outcome of interest, we made some minor amendments to our registered record and written protocol. The selection of outcomes was based on the two Core Outcome Sets of COVID-19 (Jin X. et al., 2020; Qiu R. et al., 2020) and advice of doctors participating in the treatment of COVID-19 in Wuhan.

#### Primary Outcomes

The primary outcomes of this study were improved clinical cure and the negativity of the SARS-CoV-2 nucleic acid test.

Clinical cure was defined according to the following criteria: recovery of body temperature for more than 3 days, symptom recovery, marked improvement in chest CT images, and two consecutive negative SARS-CoV-2 nucleic acid tests (at least 1 day apart) (National Health Commission of the People’s Republic of China, 2020b).

#### Secondary Outcomes

Secondary outcomes of this study included the following: 1) clinical deterioration, 2) incidence of unfavorable clinical events of acute respiratory distress syndrome (ARDS), mechanical ventilation, and intensive care unit (ICU) admission, 3) death, 4) time to fever clearance, 5) duration of hospitalization, and 6) chest imaging improvement. Clinical deterioration was defined as the progression of clinical classification (from the status at randomization), which includes ① from a mild case to moderate, severe, or critical case; ② from a moderate case to a severe or critical case;③ from a severe case to a critical case. The definition of clinical classification was defined by NHS’s protocol (National Health Commission of the People's Republic of China, 2020b), as follows: ① mild cases: mild clinical symptoms without signs of pneumonia on imaging; ② moderate cases: fever and respiratory symptoms with radiological findings of pneumonia; ③ severe cases: respiratory distress (≧30 breaths/min), oxygen saturation ≤93% at rest, arterial partial pressure of oxygen (PaO2)/fraction of inspired oxygen (FiO2) ≦ 300 mmHg (l mmHg = 0.133 kPa), lesion progression within 24–48 h > 50% on chest imaging; ④ critical cases: respiratory failure requiring mechanical ventilation and shock, with other organ failures that require ICU care.

#### Safety Outcomes

Safety outcomes included adverse events (AEs) and serious AEs, defined by the International Conference on Harmonization-Good Clinical Practice (ICH-GCP) guidelines (International Conference on Harmonisation of technical requirements for registration of pharmaceuticals for human use, 2015), that occurred during treatment. The terminologies and severity of AEs according to the Common Terminology Criteria for Adverse Events (CTCAE) (U.S. Department of Health and Human Services, 2017) and any other criterion will be included.

### Quality Assessment

The Risk of Bias 2 Tool was used to assess the methodological quality of the included studies (Sterne et al., 2019). We evaluated outcomes of the included studies of the risk of bias of the randomization process, deviation from intended intervention, missing outcome data, outcome measurement, and selection of the reported result. A low risk of bias in all five domains will lead to a low risk of overall bias. The RCTs of low risk of overall bias will be included in the meta-analysis; RCTs of unclear and high risk of overall bias will be included in the descriptive analysis.

### Evidence Synthesis for Randomized Controlled Trials

Meta-analysis was carried out when adequate data of primary and secondary outcomes were obtained, the results among the studies were homogeneous, and forest plots were presented. The mean differences (MD) for continuous data and risk ratio (RR) for dichotomous data with 95% confidence intervals (CIs) were evaluated. The random-effects model was used when synthesizing data for the meta-analysis. We quantified inconsistency by applying the *I* ^2^ statistic; a value of *I* ^2^ > 50% was considered substantial heterogeneity (Higgins et al., 2019). Subgroup and sensitivity analyses were performed to explore the source of heterogeneity if substantial heterogeneity existed. Stata 16 was used in data synthesis to perform a meta-analysis. Meta-analysis was precluded in some conditions (limited evidence for comparison or different effect measures) (Higgins et al., 2019), and descriptive analysis was used in these conditions.

#### Publication Bias

Publication bias of the cumulative evidence among individual studies was evaluated using a graphical method of funnel plot and the Egger test (Egger et al., 1997) if at least ten studies were included for the synthesized outcome.

### Quality of Evidence

The quality of the cumulative evidence was evaluated using the Grading of Recommendations Assessment, Development, and Evaluation (GRADE) system (Guyatt et al., 2008). The risk of bias, inconsistency, indirectness, imprecision, and publication bias were evaluated. Quality of evidence was classified as high, moderate, low, or very low (Guyatt et al., 2008). We presented our findings in a Summary of Finding (SoF) table. Risk difference (RD) was used to interpret the effect of TCM treatment (Poole et al., 2015; Zhang et al., 2018).

## Results

### Included Studies

**FIGURE 1:**
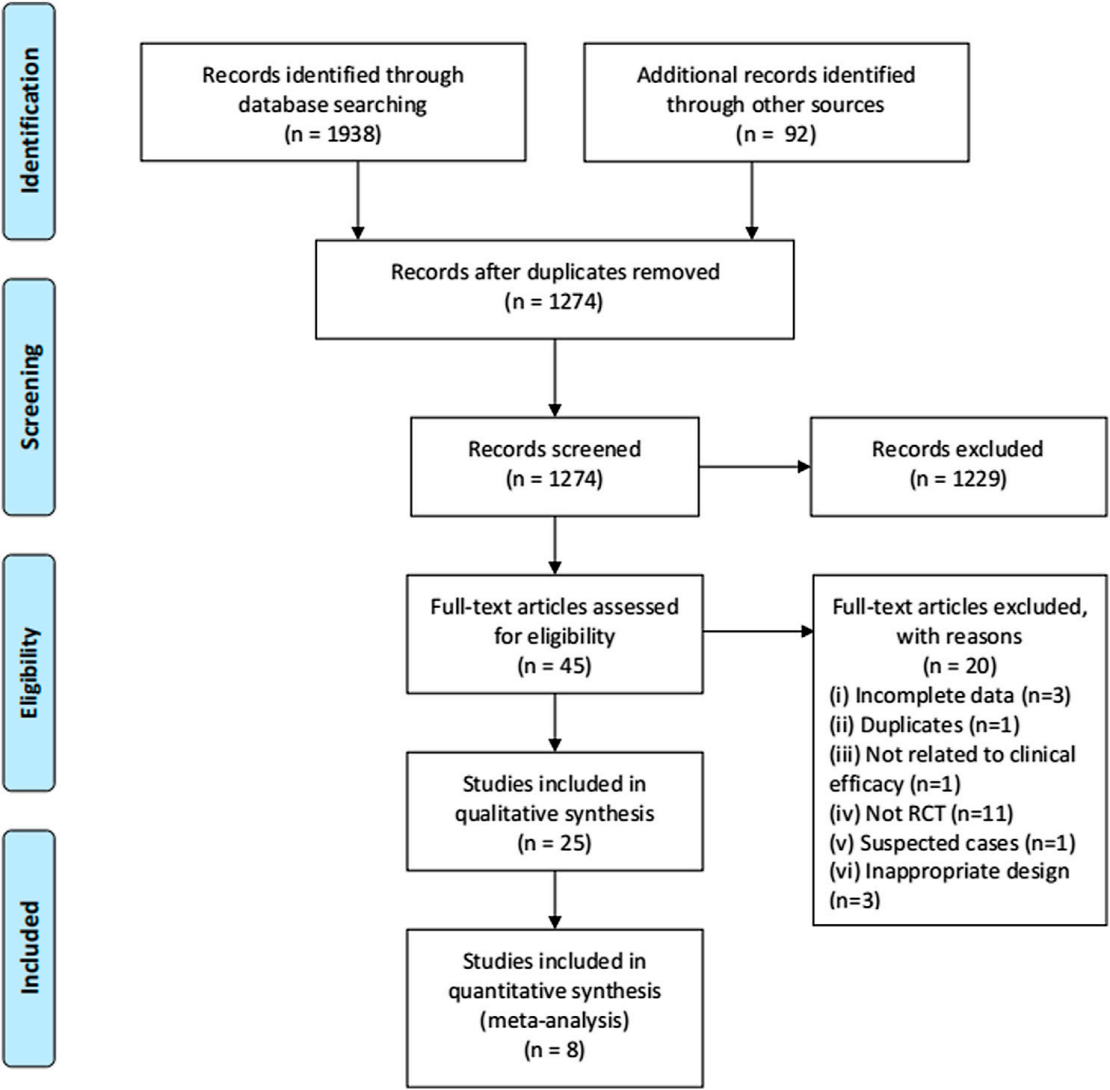
Flowchart of study selection.

The process of study selection is shown in Figure 1. A total of 25 RCTs (Ai et al., 2020; Chen et al., 2020; Ding et al., 2020; Fu et al., 2020; Hu et al., 2020; Li and Zhang, 2020; Lin et al., 2020; Qiu M. et al., 2020; Sun H. M. et al., 2020; Wang J.-b. et al., 2020; Wen et al., 2020; Ye and CHAMPS Collaborative Group, 2020; Xiong W.-z. et al., 2020; Yu et al., 2020; Zhang C. T. et al., 2020; Zhang Y. L. et al., 2020; Zhao et al., 2020a; Zheng et al., 2020; Zhou W. M. et al., 2020; Chen et al., 2021; Duan et al., 2021; He and Zhang, 2021a; Liu W. et al., 2021; Luo et al., 2021; Wang et al., 2021) with 2,222 participants were selected in our SR and seven trials were included in quantitative synthesis (Fu et al., 2020; Hu et al., 2020; Wang J.-b. et al., 2020; Wen et al., 2020; Yu et al., 2020; Zheng et al., 2020; Luo et al., 2021). Of the included trials, 24 were open-labeled RCTs, and one trial was a double-blinded RCT (Luo et al., 2021). All of the trials were conducted in mainland China, 19 of which were published in Chinese and six in English (Hu et al., 2020; Wang J.-b. et al., 2020; Xiong W.-z. et al., 2020; Ye and CHAMPS Collaborative Group, 2020; Zhao et al., 2020a; Luo et al., 2021). There were four multi-center RCTs (Hu et al., 2020; Li and Zhang, 2020; Sun H. M. et al., 2020; Zheng et al., 2020) and 20 single-center RCTs and one trial that did not mention the location of the trials (Zhang Y. L. et al., 2020). Five RCTs were registered in the Chinese Clinical Trial Registry (Hu et al., 2020; Wen et al., 2020; Xiong W.-z. et al., 2020; Ye and CHAMPS Collaborative Group, 2020; Luo et al., 2021) and one in ClinicalTrials.gov (Wang J.-b. et al., 2020). We searched the ChiCTR and ClinicalTrials.gov but found no additional records.

Details of selected RCTs are shown in Tables 1, 2. The composition of formulation and patented drugs are shown in Table 3. The course of treatment was 5–21 days, and the follow-up time was 5–29 days. The intervention groups of all 25 trials received TCM treatment plus routine treatment. The efficacy of the TCM formula was evaluated in 16 trials, six trials evaluated oral Chinese patented drugs (Hu et al., 2020; Sun H. M. et al., 2020; Yu et al., 2020; Zhang Y. L. et al., 2020; Chen et al., 2021; Duan et al., 2021), three trials evaluated Chinese medicine injection of Xuebijing (Chen et al., 2020; Wen et al., 2020; Luo et al., 2021), one trial evaluated Chinese medicine extracts (Zhou W. M. et al., 2020), and one trial evaluated the clinical efficacy of the TCM formula and oral Chinese patented drugs (Liu W. et al., 2021). Control groups received routine treatment recommended by the Diagnosis and Treatment Protocol of Coronavirus Disease 2019, which includes antiviral treatment (alpha interferon inhalation, lopinavir/ritonavir, ribavirin, and Arbidol), antibacterial treatment, oxygen therapy, and supportive treatment (National Health Commission of the People’s Republic of China, 2020b).

**TABLE 1:** Study design, population’s details, and outcome of selected studies.

Study	Study design	Sample size		Age		Sex (male/female)		Clinical classification		Outcome
								(mild/moderate/severe/critical)		
		TCM + RT	RT	TCM + RT	RT	TCM + RT	RT	TCM + RT	RT	
Ding et al. (2020)	Single-center	51	49	54.7 ± 21.3	50.8 ± 23.5	39/12	39/10	10/36/5/0	11/34/4/0	⑥⑩
Duan et al. (2021)	Single-center	82	41	51.99 ± 13.88	50.29 ± 13.17	39/43	23/18	82/0/0/0	41/0/0/0	③⑩
Fu et al. (2020)	Single-center	37	36	45.26 ± 7.25	44.68 ± 7.45	19/18	19/17	0/37/0/0	0/36/0/0	①③⑩
Li and Zhang (2020)	Multi-center	6	6	52.00 ± 6.56	50.00 ± 10.00	2/4	3/3	0/0/6/0	0/0/6/0	①⑧⑩
Qiu et al. (2020b)	Single-center	25	25	53.35 ± 18.35	51.32 ± 14.62	13/12	14/11	0/25/0/0	0/25/0/0	③⑥⑨
Sun et al. (2020b)	Multi-center	32	25	45.4 ± 14.1	42.0 ± 11.7	17/15	11/14	4/28/0/0	3/22/0/0	③⑥
Wen et al. (2020)	Single-center	20	20	47.1 ± 5.2	47.7 ± 5.7	12/8	9/11	0/0/20/0	0/0/20/0	②③⑩
Yu et al. (2020)	Single-center	147	148	48.27 ± 9.56	47.25 ± 8.67	82/65	89/59	14/133/0/0	13/135/0/0	③⑥⑦⑩
Zheng et al. (2020)	Multi-center	65	65	17–84	18–85	42/23	44/21	0/0/59/6	0/0/60/5	①⑦
Zhou et al. (2020b)	Single-center	52	52	52.47 ± 10.99	51.11 ± 9.87	32/20	28/24	0/52/0/0	0/52/0/0	①③⑩
Hu et al. (2020)	Multi-center	142	142	50.4 ± 15.2	51.8 ± 14.8	79/63	71/71	—	—	①②③⑥⑩
Wang et al. (2020b)	Single-center	24	23	46.8 ± 14.4	51.4 ± 17.6	14/10	12/11	—	—	②④⑤⑥⑦⑨⑩
Ye and CHAMPS Collaborative Group, (2020)	Single-center	28	14	65 (53.5–69)	59 (47–67)	2/25	4/10	—	—	①③⑤⑥⑦
Zhao et al. (2020a)	Single-center	15	24	—	—	8/7	14/10	0/0/15/0	0/0/24/0	①⑧⑨
Ai et al. (2020)	Single-center	55	43	43.98 ± 12.6	45.95 ± 18.3	24/31	17/26	8/40/7/0	6/33/4/0	⑧⑩
Chen et al. (2021)	Single-center	30	30	50.16 ± 5.11	49.52 ± 5.06	17/13	18/12	—	—	③⑨⑩
Chen et al. (2020)	Single-center	15	15	42.6 ± 3.5	43.1 ± 3.2	8/7	9/6	—	—	①⑩
He and Zhang, (2021a)	Single-center	34	30	—	—	—	—	—	—	②⑥
Lin et al. (2020)	Single-center	41	41	46.02 ± 12.09	43.80 ± 12.34	15/26	23/18	0/41/0/0	0/41/0/0	③⑥⑧⑩
Liu et al. (2021b)	Single-center	44	44	48.51 ± 4.56	48.43 ± 4.52	16/28	15/29	44/0/0/0	44/0/0/0	①⑩
Wang et al. (2021)	Single-center	70	70	48.0 ± 13.2	49.4 ± 13.3	35/35	36/34	0/70/0/0	0/70/0/0	⑥⑧⑩
Zhang et al. (2020c)	Single-center	22	23	53.7 ± 3.5	55.6 ± 4.2	9/13	10/13	0/22/0/0	0/23/0/0	⑥⑩
Zhang et al. (2020d)	—	80	40	53.4 ± 13.7	52.0 ± 14.1	50/30	23/17	0/80/0/0	0/40/0/0	③⑩
Luo et al. (2021)	Single-center	29	28	60.26 ± 15.62	56.35 ± 18.28	—	—	0/0/29/0	0/0/28/0	③④⑤⑦⑨
Xiong et al. (2020b)	Single-center	22	20	57.10 ± 14.00	62.40 ± 12.30	—	—	—	—	⑩

① Clinical cure, ② negativity of SARS-CoV-2 nucleic acid test, ③ clinical deterioration, ④ ARDS, ⑤ mechanical ventilation, ⑥ chest image improvement, ⑦ death, ⑧ duration of hospitalization, ⑨ time to fever clearance, and ⑩ adverse events.

TCM, traditional Chinese medicine; RT, routine treatment.

**TABLE 2:** Intervention details of selected studies.

Study	Intervention		Course of Treatment
	Routine treatment	TCM plus routine treatment	
Ding et al. (2020)	Antivirus treatment (alpha interferon inhalation, 50 μg twice daily; ribavirin 0.5 g intravenously twice daily), antibacterial treatment, and oxygen therapy for severe cases	The same treatments as in the control group and Qingfei Touxie Fuzheng Recipe 150 ml orally twice daily	10 days
Duan et al. (2021)	Antivirus treatment and antibacterial treatment	The same treatments as in the control group and Jinhua Qinggan Granule 10 g thrice daily	5 days
Fu et al. (2020)	Antiviral treatment (Aribidol 0.2 g orally thrice daily), and Ambroxol hydrochloride 30 mg orally thrice daily	The same treatments as in the control group and Toujie Quwen granules one portion twice daily	15 days
Li and Zhang (2020)	Supportive treatments, antiviral treatment (alpha interferon inhalation and ribavirin), and antibacterial treatment	The same treatments as in the control group and Qingfei Paidu Decoction, one unit of decoction divided into two portions, one portion orally twice daily	6 days
Qiu et al. (2020b)	Antiviral treatment: alpha interferon inhalation, 50 μg twice daily; and lopinavir/ritonavir, 400 mg/100 mg twice daily	Antiviral treatment and Maxing Xuanfei Jiedu decoction, 150 ml thrice daily	10 days
Sun et al. (2020b)	Supportive treatments and antiviral treatment (alpha interferon inhalation and lopinavir/ritonavir)	The same treatments as in the control group and Lianhua Qingke granule one unit thrice daily	14 days
Wen et al. (2020)	Routine treatment recommended by the COVID-19 Diagnosis and Treatment Protocol	Routine treatment as in the control group and Xuebijing injection 100 ml intravenously, twice daily	7 days
Yu et al. (2020)	Antiviral treatment (Aribidol 0.2 g orally thrice daily), antibacterial treatment (moxifloxacin 0.4 g orally once daily), and Ambroxol hydrochloride 30 mg orally thrice daily	The same treatments as in the control group and Lianhua Qingwen Capsules, 6 g thrice daily	7 days
Zheng et al. (2020)	Supportive treatments, antiviral treatment (alpha interferon inhalation, lopinavir/ritonavir, and Aribidol), antibacterial treatment (moxifloxacin), and methylprednisolone	The same treatments as in the control group and TCM formula, one unit of formula yielded 300 ml, divided into three portions, one portion orally thrice daily	14 days
Zhou et al. (2020b)	Supportive treatments and antiviral treatment (lopinavir/ritonavir, 500 mg twice daily)	The same treatments as in the control group and diammonium glycyrrhizinate capsules (three capsules thrice daily)	2 weeks
Hu et al. (2020)	Supportive treatment such as oxygen therapy, antiviral medications and symptomatic therapies	Supportive treatment and Lianhua Qingwen Capsules (four capsules thrice daily)	14 days
Wang et al. (2020b)	Supportive treatments and antiviral treatment (alpha interferon inhalation, 50 μg twice daily; and lopinavir/ritonavir, 400 and 100 mg twice daily, respectively)	The same treatments as in the control group and Keguan-1 19.4 g twice daily	14 days
Ye and CHAMPS Collaborative Group, (2020)	Standard care: supplementary oxygen, intravenous fluids, and routine pharmaceutical medications. Ribavirin/Arbidole was part of the standard care in China	Standard care as in the control group and TCM formula, one unit of formula yielded 400 ml of decoction, divided into two portions, one portion orally twice daily	7 days
Zhao et al. (2020a)	General treatment: bed rest and supportive treatments; ensuring sufficient calories and water intake; maintaining water-electrolyte balance and homeostasis	General treatment as in the control group and TCM prescription orally	2 weeks
Ai et al. (2020)	Antiviral therapy such as abidol, lopinavir, tolonavir or chloroquine, and symptomatic treatment such as oxygen therapy, anti-inflammatory, and expectorant treatment	The same treatments as in the control group and TCM granules of “Pneumonia No.1 Prescription,” 100 ml orally twice daily	12 days
Chen et al. (2021)	General treatment such as bed rest, supportive treatments, ensuring sufficient calories and water intake. Antiviral treatment (alpha interferon aerosol inhalation, 5 million IU with 2 ml sterile water, inhalation twice daily; lopinavir/ritonavir, two tablets orally twice daily)	The same treatments as in the control group and Lianhua Qingwen capsule, four capsules, orally thrice daily	10 days
Chen et al. (2020)	Supportive treatment and antiviral treatment such as alpha interferon aerosol inhalation and lopinavir/ritonavir orally	The same treatments as in the control group and 100 ml of Xuebijing injection with 250 ml NS, intravenous drip, twice daily	2 weeks
He and Zhang (2021a)	Symptomatic supportive treatment and antiviral treatment recommended by 6th edition protocol	The same treatments as in the control group and Shengmai San, orally twice daily, modified according to syndrome differentiation	7 days
Lin et al. (2020)	General treatment such as bed rest, supportive treatments, ensuring sufficient calories and water intake. Antiviral treatment (alpha interferon aerosol inhalation, 5 million IU with 2 ml sterile water, inhalation twice daily; lopinavir/ritonavir, two tablets orally twice daily)	The same treatments as in the control group and Xuanfei Qingre recipe, 150 ml orally twice daily	14 days
Liu et al. (2020)	Antiviral treatment (Aribidol 0.2 g orally thrice daily, Oseltamivir 15 mg orally twice daily) and supportive treatment	The same treatments as in the control group and Lianhua Qingwen capsule, 1.4 g, orally thrice daily. “Pneumonia No.2 Prescription,” one unit of decoction divided into two portions, one portion twice daily	21 days
Wang et al. (2021)	Supportive treatments, antiviral treatment (Aribidol 0.2 g orally thrice daily), antibacterial treatment (moxifloxacin 0.4 g orally once daily)	The same treatments as in the control group and TCM granules of Qingfei Paidu Decoction, 100 ml orally twice daily	10 days
Zhang et al. (2020c)	Supportive treatment and antiviral treatment recommended by 4th edition protocol	The same treatments as in the control group and TCM granules of Dayuan Decoction, one unit of decoction divided into two portions, one portion twice daily	7 days
Zhang et al. (2020d)	Supportive treatment and treatment (alpha interferon inhalation, 5 million U with 2 ml sterile water, inhalation twice daily; lopinavir/ritonavir, two tablets orally twice daily)	The same treatments as in the control group and Jinyinhua Oral liquid, 60 ml, thrice daily	10 days
Luo et al. (2021)	Supportive treatment, antiviral treatment (alpha interferon inhalation), antibiotic agents, noninvasive and invasive ventilation if necessary.150 ml NS, intravenous drip, every 12 h	The same treatments as in the control group and 50 ml XBJ injection diluted with 100 ml NS, intravenous drip, every 12 h	14 days
Xiong et al. (2020b)	Routine treatment recommended by the COVID-19 Diagnosis and Treatment Protocol	Routine treatment and Xuanfei Baidu decoction 200 ml, orally twice daily	1 week

**TABLE 3:** Composition of formulation and patented drugs.

Study	Formulation or patented drugs	Source	Composition	Quality control reported?	Chemical analysis reported?
Ding et al. (2020)	Qingfei Touxie Fuzheng Recipe	—	*Ephedra sinica* Stapf [Ephedraceae; Ephedrae herba praeparata cum melle] 6 g, Gypsum Fibrosum 20 g, *Prunus armeniaca* L. [Rosaceae; Armeniacae semen amarum] 10 g, Lonicera japonica Thunb. [Caprifoliaceae; *Lonicerae japonicae* flos] 30 g, *Forsythia suspensa* (Thunb.) Vahl [Oleaceae; Forsythiae fructus] 15 g, *Phragmites australis* (Cav.) Trin. ex Steud. [Poaceae; Phragmitis rhizoma] 30 g, *Coix lacryma-jobi var. ma-yuen* (Rom.Caill.) Stapf [Poaceae; Coicis semen] 30 g, body of sick *Bombyx mori* Linnaeus [Bombycidae; Bombyx batryticatus] 10 g, *Cryptotympana pustulata* Fabricius [Cicadidae; Cicadae periostracum] 10 g, *Reynoutria japonica* Houtt. [Polygonaceae; Polygoni cuspidati rhizoma et radix] 15 g, *Curcuma longa* L. [Zingiberaceae; Curcumae longae rhizoma] 10 g, *Paeonia lactiflora* Pall. [Paeoniaceae; Paeoniae radix alba] 10 g, *Pseudostellaria heterophylla* (Miq.) Pax [Caryophyllaceae; Pseudostellariae radix] 20 g, *Glycyrrhiza uralensis* Fisch. ex DC. [Fabaceae; Glycyrrhizae radix et rhizoma] 15 g	N	N
Duan et al. (2021)	Jinhua Qinggan granule	Beijing Juxiechang Pharmaceutical	*Lonicera japonica* Thunb. [Caprifoliaceae; Lonicerae japonicae flos], Gypsum Fibrosum, *Ephedra sinica* Stapf [Ephedraceae; Ephedrae herba praeparata cum melle], *Prunus armeniaca* L. [Rosaceae; Armeniacae semen amarum], *Scutellaria baicalensis* Georgi [Lamiaceae; Scutellariae radix], *Forsythia suspensa* (Thunb.) Vahl [Oleaceae; Forsythiae fructus], *Fritillaria thunbergii* Miq. [Liliaceae; Fritillariae thunbergii bulbus], *Anemarrhena asphodeloides* Bunge [Asparagaceae; Anemarrhenae rhizoma], *Arctium lappa* L. [Asteraceae; Arctii fructus], *Artemisia annua* L. [Asteraceae; Artemisiae annuae herba], *Mentha canadensis* L. [Lamiaceae; Menthae haplocalycis herba], *Glycyrrhiza uralensis* Fisch. ex DC. [Fabaceae; Glycyrrhizae radix et rhizoma]	N	N
Fu et al. (2020)	Toujie Quwen granules	Guangdong E-fong Pharmaceutical	*Forsythia suspensa* (Thunb.) Vahl [Oleaceae; Forsythiae fructus] 30 g, *Cremastra appendiculata* (D.Don) Makino [Orchidaceae; Cremastrae pseudobulbus pleiones pseudobulbus] 20 g, *Lonicera japonica* Thunb. [Caprifoliaceae; Lonicerae japonicae flos] 15 g, *Scutellaria baicalensis* Georgi [Lamiaceae; Scutellariae radix] 10 g, *Isatis tinctoria* L. [Brassicaceae; Isatidis folium] 10 g, *Bupleurum chinense* DC. [Apiaceae; Bupleuri radix] 5 g, *Artemisia annua* L. [Asteraceae; Artemisiae annuae herba] 10 g, *Cryptotympana pustulata* Fabricius [Cicadidae; Cicadae periostracum] 10 g, *Kitagawia praeruptora* (Dunn) Pimenov [Apiaceae; Peucedani radix] 5 g, *Fritillaria cirrhosa* D.Don [Liliaceae; Fritillariae cirrhosae bulbus] 10 g, *Fritillaria thunbergii* Miq. [Liliaceae; Fritillariae thunbergii bulbus] 10 g, *Prunus mume* (Siebold) Siebold and Zucc. [Rosaceae; Mume fructus] 30 g, *Scrophularia ningpoensis* Hemsl. [Scrophulariaceae; Scrophulariae radix] 10 g, *Astragalus mongholicus* Bunge [Fabaceae; Astragali radix] 45 g, *Poria cocos* (Schw.) Wolf [Polyporaceae; Poria] 30 g, *Pseudostellaria heterophylla* (Miq.) Pax [Caryophyllaceae; Pseudostellariae radix] 15 g	N	N
Li and Zhang (2020)	Qingfei Paidu Decoction	-	*Ephedra sinica* Stapf [Ephedraceae; Ephedrae herba] 9 g, *Glycyrrhiza uralensis* Fisch. ex DC. [Fabaceae; Glycyrrhizae radix et rhizoma praeparata cum melle] 6 g, *Prunus armeniaca* L. [Rosaceae; Armeniacae semen amarum] 9 g, Gypsum Fibrosum 15–30 g, *Cinnamomum cassia* (L.) J.Presl. [Lauraceae; Cinnamomi ramulus] 9 g, *Alisma plantago-aquatica* L. [Alismataceae; Alismatis rhizoma] 9 g, *Polyporus umbellatus* (Pers.) Fries [Polyporaceae; Polyporus] 9 g, *Atractylodes macrocephala* Koidz. [Asteraceae; Atractylodis macrocephalae rhizoma] 9 g, *Poria cocos* (Schw.) Wolf [Polyporaceae; Poria] 15 g, *Bupleurum chinense* DC. [Apiaceae; Bupleuri radix] 16 g, *Scutellaria baicalensis* Georgi [Lamiaceae; Scutellariae radix] 6 g, *Pinellia ternata* (Thunb.) Makino [Araceae; Pinelliae rhizoma praeparatum cum zingibere et alumine] 9 g, *Zingiber officinale* Roscoe [Zingiberaceae; Zingiberis rhizoma recens] 9 g, *Aster tataricus* L.f. [Asteraceae; Asteris radix et rhizoma] 9 g, *Tussilago farfara* L. [Asteraceae; Farfarae flos] 9 g, *Iris domestica* (L.) Goldblatt and Mabb. [Iridaceae; Belamcandae rhizoma] 9 g, *Asarum sieboldii* Miq. [Aristolochiaceae; Asari radix et rhizoma] 6 g, *Dioscorea oppositifolia* L. [Dioscoreaceae; Dioscoreae rhizoma] 12 g, *Citrus × aurantium* L. [Rutaceae; Aurantii fructus immaturus] 6 g, *Citrus reticulata* Blanco [Rutaceae; Citri reticulatae pericarpium] 6 g, *Pogostemon cablin* (Blanco) Benth. [Lamiaceae; Pogostemonis herba] 9 g	N	N
Qiu et al. (2020b)	Maxing Xuanfei Jiedu Decoction	Pharmacy of Chongqing Traditional Chinese Medicine Hospital	*Ephedra sinica* Stapf [Ephedraceae; Ephedrae herba] 9 g, *Prunus armeniaca* L. [Rosaceae; Armeniacae semen amarum] 12 g, Gypsum Fibrosum 15–30 g, *Fritillaria thunbergii* Miq. [Liliaceae; Fritillariae thunbergii bulbus] 12 g, *Cryptotympana pustulata* Fabricius [Cicadidae; Cicadae periostracum] 10 g, body of sick *Bombyx mori* Linnaeus [Bombycidae; Bombyx batryticatus] 15 g, *Curcuma longa* L. [Zingiberaceae; Curcumae longae rhizoma] 12 g, *Platycodon grandiflorus* (Jacq.) A.DC. [Campanulaceae; Platycodonis radix] 12 g, *Citrus × aurantium* L. [Rutaceae; Aurantii fructus] 12 g, *Amomum tsao-ko* Crevost and Lemarié [Zingiberaceae; Tsaoko fructus] 9 g, *Amomurn kravanh* Pierre ex Gagnep. [Zingiberaceae; Amomi fructus rotundus] 12 g	N	N
Sun et al. (2020b)	Lianhua Qingke granule	Shijiazhuang Yiling Pharmaceutical	*Ephedra sinica* Stapf [Ephedraceae; Ephedrae herba], Gypsum Fibrosum, *Forsythia suspensa* (Thunb.) Vahl [Oleaceae; Forsythiae fructus], *Scutellaria baicalensis* Georgi [Lamiaceae; Scutellariae radix], *Morus alba* L. [Moraceae; Mori cortex], *Prunus armeniaca* L. [Rosaceae; Armeniacae semen amarum], *Kitagawia praeruptora* (Dunn) Pimenov [Apiaceae; Peucedani radix], *Pinellia ternata* (Thunb.) Makino [Araceae; Pinelliae rhizoma praeparatum cum alumine], *Citrus reticulata* Blanco [Rutaceae; Citri reticulatae pericarpium], *Fritillaria thunbergii* Miq. [Liliaceae; Fritillariae thunbergii bulbus], *Arctium lappa* L. [Asteraceae; Arctii fructus], *Lonicera confusa* DC. [Caprifoliaceae; Lonicerae flos], *Rheum palmatum* L. [Polygonaceae; Rhei radix et rhizoma], *Platycodon grandiflorus* (Jacq.) A.DC. [Campanulaceae; Platycodonis radix], *Glycyrrhiza uralensis* Fisch. ex DC. [Fabaceae; Glycyrrhizae radix et rhizoma]	N	N
Wen et al. (2020)	Xuebijing injection	Tianjin Chase Sun Pharmaceutical	*Carthamus tinctorius* L. [Asteraceae; Carthami flos], *Paeonia lactiflora* Pall. [Paeoniaceae; Paeoniae radix rubra], *Ligusticum striatum* DC. [Apiaceae; Chuanxiong rhizoma], *Salvia miltiorrhiza* Bunge [Lamiaceae; Salviae miltiorrhizae radix et rhizoma], *Angelica sinensis* (Oliv.) Diels [Apiaceae; Angelicae sinensis radix]	N	N
Yu et al. (2020)	Lianhua Qingwen granule	Beijing Yiling Pharmaceutical	*Forsythia suspensa* (Thunb.) Vahl [Oleaceae; Forsythiae fructus], *Lonicera japonica* Thunb. [Caprifoliaceae; Lonicerae japonicae flos], *Ephedra sinica* Stapf [Ephedraceae; Ephedrae herba praeparata cum melle], *Prunus armeniaca* L. [Rosaceae; Armeniacae semen amarum], Gypsum Fibrosum, *Isatis tinctoria* L. [Brassicaceae; Isatidis radix], *Dryopteris crassirhizoma* Nakai [Polypodiaceae; Dryopteridis crassirhizomatis rhizoma], *Houttuynia cordata* Thunb. [Saururaceae; Houttuyniae herba], *Pogostemon cablin* (Blanco) Benth. [Lamiaceae; Pogostemonis herba], *Rheum palmatum* L. [Polygonaceae; Rhei radix et rhizoma], *Rhodiola crenulata* (Hook.f. and Thomson) H.Ohba [Crassulaceae; Rhodiolae crenulatae radix et rhizoma], 1-menthol, Glycyrrhiza uralensis Fisch. ex DC. [Fabaceae; Glycyrrhizae radix et rhizoma]	N	N
Zheng et al. (2020)	Xiaochaihu Decoction and Maxing Shigan Decoction	—	Xiaochaihu Decoction and Maxing Shigan Decoction: *Bupleurum chinense* DC. [Apiaceae; Bupleuri radix] 20 g, *Scutellaria baicalensis* Georgi [Lamiaceae; Scutellariae radix] 12 g, *Pinellia ternata* (Thunb.) Makino [Araceae; Pinelliae rhizoma praeparatum] 12 g, *Codonopsis pilosula* (Franch.) Nannf. [Campanulaceae; Codonopsis radix] 15 g, *Zingiber officinale* Roscoe [Zingiberaceae; Zingiberis rhizoma] 10 g, *Ziziphus jujuba* Mill. [Rhamnaceae; Jujubae fructus] 12 g, *Glycyrrhiza uralensis* Fisch. ex DC. [Fabaceae; Glycyrrhizae radix et rhizoma praeparata cum melle] 10 g, *Ephedra sinica* Stapf [Ephedraceae; Ephedrae herba] 10 g, *Prunus armeniaca* L. [Rosaceae; Armeniacae semen amarum] 12 g, Gypsum Fibrosum 30 g, *Phragmites australis* (Cav.) Trin. ex Steud. [Poaceae; Phragmitis rhizoma] 30 g, *Aster tataricus* L.f. [Asteraceae; Asteris radix et rhizoma] 15 g, *Tussilago farfara* L. [Asteraceae; Farfarae flos] 15 g, *Cryptotympana pustulata* Fabricius [Cicadidae; Cicadae periostracum] 10 g, *Coix lacryma-jobi var. ma-yuen* (Rom.Caill.) Stapf [Poaceae; Coicis semen] 20 g, *Hordeum vulgare* L. [Poaceae; Hordei fructus germinatus] 20 g	N	N
			—		
	Modified Sanren Decoction		Modified Sanren Decoction: *Prunus armeniaca* L. [Rosaceae; Armeniacae semen amarum] 10 g, *Amomurn kravanh* Pierre ex Gagnep. [Zingiberaceae; Amomi fructus rotundus] 10 g, *Coix lacryma-jobi var. ma-yuen* (Rom.Caill.) Stapf [Poaceae; Coicis semen] 30 g, *Magnolia officinalis* Rehder and E.H.Wilson [Magnoliaceae; Magnoliae officinalis cortex] 10 g, *Pinellia ternata* (Thunb.) Makino [Araceae; Pinelliae rhizoma praeparatum] 10 g, *Tetrapanax papyrifer* (Hook.) K.Koch [Araliaceae; Tetrapanacis medulla] 10 g, *Glycyrrhiza uralensis* Fisch. ex DC. [Fabaceae; Glycyrrhizae radix et rhizoma] 10 g, Talci pulvis 10 g, *Anemarrhena asphodeloides* Bunge [Asparagaceae; Anemarrhenae rhizoma] 10 g, *Scutellaria baicalensis* Georgi [Lamiaceae; Scutellariae radix] 10 g, *Ephedra sinica* Stapf [Ephedraceae; Ephedrae herba] 8 g, *Poria cocos* (Schw.) Wolf [Polyporaceae; Poria] 10 g, *Bupleurum chinense* DC. [Apiaceae; Bupleuri radix] 15 g, *Lophatherum gracile* Brongn. [Poaceae; Lophatheri herba] 10 g		
Zhou et al. (2020b)	Diammonium Glycyrrhizinate Capsules	Chia Tai TianQing Pharmaceutical	Diammonium glycyrrhizinate 50 mg	N	N
Hu et al. (2020)	Lianhua Qingwen Capsules	Shijiazhuang Yiling Pharmaceutical	*Forsythia suspensa* (Thunb.) Vahl [Oleaceae; Forsythiae fructus], *Lonicera japonica* Thunb. [Caprifoliaceae; Lonicerae japonicae flos], *Ephedra sinica* Stapf [Ephedraceae; Ephedrae herba praeparata cum melle], *Prunus armeniaca* L. [Rosaceae; Armeniacae semen amarum], Gypsum Fibrosum, *Isatis tinctoria* L. [Brassicaceae; Isatidis radix], *Dryopteris crassirhizoma* Nakai [Polypodiaceae; Dryopteridis crassirhizomatis rhizoma], *Houttuynia cordata* Thunb. [Saururaceae; Houttuyniae herba], *Pogostemon cablin* (Blanco) Benth. [Lamiaceae; Pogostemonis herba], *Rheum palmatum* L. [Polygonaceae; Rhei radix et rhizoma], *Rhodiola crenulata* (Hook.f. and Thomson) H.Ohba [Crassulaceae; Rhodiolae crenulatae radix et rhizoma], 1-menthol, *Glycyrrhiza uralensis* Fisch. ex DC. [Fabaceae; Glycyrrhizae radix et rhizoma]	Y, prepared according to *The Pharmacopeia of People’s Republic of China*	N
Wang et al. (2020b)	Keguan-1	Beijing Tcmages Pharmaceutical	*Lonicera japonica* Thunb. [Caprifoliaceae; Lonicerae japonicae flos] 30 g, *Forsythia suspensa* (Thunb.) Vahl [Oleaceae; Forsythiae fructus] 30 g, *Morus alba* L. [Moraceae; Mori folium] 15 g, *Chrysanthemum × morifolium* (Ramat.) Hemsl. [Asteraceae; Chrysanthemi flos] 10 g, *Coix lacryma-jobi var. ma-yuen* (Rom.Caill.) Stapf [Poaceae; Coicis semen] 30 g, *Fritillaria thunbergii* Miq. [Liliaceae; Fritillariae thunbergii bulbus] 15 g, *Prunus armeniaca* L. [Rosaceae; Armeniacae semen amarum] 9 g	N	Y, HPLC
Ye and CHAMPS Collaborative Group, (2020)	Modified Maxingshigan Formula;	Jiangyin Tianjiang Pharmaceutical	Modified Maxingshigan Formula: *Prunus armeniaca* L. [Rosaceae; Armeniacae semen amarum] 10 g, Gypsum Fibrosum 30 g, *Trichosanthes kirilowii* Maxim. [Cucurbitaceae; Trichosanthis fructus] 30 g, *Rheum palmatum* L. [Polygonaceae; Rhei radix et rhizoma] 6 g (added at the end of decoction preparation), *Ephedra sinica* Stapf [Ephedraceae; Ephedrae herba] 6 g, *Ephedra sinica* Stapf [Ephedraceae; Ephedrae herba praeparata cum melle] 6 g, *Descurainia sophia* (L.) Webb ex Prantl [Brassicaceae; Descurainiae semen] 10 g, *Prunus persica* (L.) Batsch [Rosaceae; Persicae semen] 10 g, *Amomum tsao-ko* Crevost and Lemarié [Zingiberaceae; Tsaoko fructus] 6 g, *Areca catechu* L. [Arecaceae; Arecae semen] 10 g, *Atractylodes lancea* (Thunb.) DC. [Asteraceae; Atractylodis rhizoma] 10 g;	Y, prepared according to 2015 Chinese Pharmacopoeia	N
	Modified Shenfutang formula		Modified Shenfutang Formula: *Panax ginseng* C.A.Mey. [Araliaceae; Ginseng Radix et Rhizoma] 15 g, *Aconitum carmichaelii* Debeaux [Ranunculaceae; Aconiti lateralis radix praeparata] 10 g (cook prior to mixture with other herbs); *Cornus officinalis* Siebold and Zucc. [Cornaceae; Corni fructus] 15 g		
Zhao et al. (2020a)	Yidu-toxicity Blocking Lung Decoction	Guangdong E-fong Pharmaceutical	*Prunus armeniaca* L. [Rosaceae; Armeniacae semen amarum] 10 g, Gypsum Fibrosum 30 g, *Trichosanthes kirilowii* Maxim. [Cucurbitaceae; Trichosanthis fructus] 30 g, *Rheum palmatum* L. [Polygonaceae; Rhei radix et rhizoma] 6 g, *Ephedra sinica* Stapf [Ephedraceae; Ephedrae herba] 6 g, *Ephedra sinica* Stapf [Ephedraceae; Ephedrae herba praeparata cum melle] 6 g, *Descurainia sophia* (L.) Webb ex Prantl [Brassicaceae; Descurainiae semen] 10 g, *Prunus persica* (L.) Batsch [Rosaceae; Persicae semen] 10 g, *Amomum tsao-ko* Crevost and Lemarié [Zingiberaceae; Tsaoko fructus] 6 g, *Areca catechu* L. [Arecaceae; Arecae semen] 10 g, *Atractylodes lancea* (Thunb.) DC. [Asteraceae; Atractylodis rhizoma] 10 g	N	N
Ai et al. (2020)	Pneumonia No.1 Formula	-	*Artemisia annua* L. [Asteraceae; Artemisiae annuae herba] 10 g, *Astragalus mongholicus* Bunge [Fabaceae; Astragali radix] 45 g, *Cremastra appendiculata* (D.Don) Makino [Orchidaceae; Cremastrae pseudobulbus pleiones pseudobulbus] 20 g, *Forsythia suspensa* (Thunb.) Vahl [Oleaceae; Forsythiae fructus] 30 g, *Scutellaria baicalensis* Georgi [Lamiaceae; Scutellariae radix] 10 g, *Lonicera japonica* Thunb. [Caprifoliaceae; Lonicerae japonicae flos] 15 g, *Isatis tinctoria* L. [Brassicaceae; Isatidis folium] 10 g, *Bupleurum chinense* DC. [Apiaceae; Bupleuri radix] 5 g, *Cryptotympana pustulata* Fabricius [Cicadidae; Cicadae periostracum] 10 g, *Kitagawia praeruptora* (Dunn) Pimenov [Apiaceae; Peucedani radix] 5 g, *Fritillaria cirrhosa* D.Don [Liliaceae; Fritillariae cirrhosae bulbus] 10 g, *Fritillaria thunbergii* Miq. [Liliaceae; Fritillariae thunbergii bulbus] 10 g, *Prunus mume* (Siebold) Siebold and Zucc. [Rosaceae; Mume fructus] 30 g, *Scrophularia ningpoensis* Hemsl. [Scrophulariaceae; Scrophulariae radix] 10 g, *Poria cocos* (Schw.) Wolf [Polyporaceae; Poria] 30 g, *Pseudostellaria heterophylla* (Miq.) Pax [Caryophyllaceae; Pseudostellariae radix] 15 g	N	N
Chen et al. (2021)	Lianhua Qingwen Capsules	Shijiazhuang Yiling Pharmaceutical	*Forsythia suspensa* (Thunb.) Vahl [Oleaceae; Forsythiae fructus], *Lonicera japonica* Thunb. [Caprifoliaceae; Lonicerae japonicae flos], *Ephedra sinica* Stapf [Ephedraceae; Ephedrae herba praeparata cum melle], *Prunus armeniaca* L. [Rosaceae; Armeniacae semen amarum], Gypsum Fibrosum, *Isatis tinctoria* L. [Brassicaceae; Isatidis radix], *Dryopteris crassirhizoma* Nakai [Polypodiaceae; Dryopteris crassirhizomatis rhizoma], *Houttuynia cordata* Thunb. [Saururaceae; Houttuyniae herba], *Pogostemon cablin* (Blanco) Benth. [Lamiaceae; Pogostemonis herba], *Rheum palmatum* L. [Polygonaceae; Rhei radix et rhizoma], *Rhodiola crenulata* (Hook.f. and Thomson) H.Ohba [Crassulaceae; Rhodiolae crenulatae radix et rhizoma], 1-menthol, *Glycyrrhiza uralensis* Fisch. ex DC. [Fabaceae; Glycyrrhizae radix et rhizoma]	—	—
Chen et al. (2020)	Xuebijing Injection	Tianjin Chase Sun Pharmaceutical	*Carthamus tinctorius* L. [Asteraceae; Carthami flos], *Paeonia lactiflora* Pall. [Paeoniaceae; Paeoniae radix rubra], *Ligusticum striatum* DC. [Apiaceae; Chuanxiong rhizoma], *Salvia miltiorrhiza* Bunge [Lamiaceae; Salviae miltiorrhizae radix et rhizoma], *Angelica sinensis* (Oliv.) Diels [Apiaceae; Angelicae sinensis radix]	N	N
He and Zhang, (2021a)	Modified Shengmaisan Formula	—	—	N	N
Lin et al. (2020)	Xuanfei Qingre Formula	—	*Ephedra sinica* Stapf [Ephedraceae; Ephedrae herba] 9 g, *Prunus armeniaca* L. [Rosaceae; Armeniacae semen amarum] 12 g, Gypsum Fibrosum 30 g (cook prior to mixture with other herbs), *Glycyrrhiza uralensis* Fisch. ex DC. [Fabaceae; Glycyrrhizae radix et rhizoma] 6 g, *Prunus persica* (L.) Batsch [Rosaceae; Persicae semen] 12 g, *Benincasa hispida* (Thunb.) Cogn. [Cucurbitaceae; Benincasae semen] 30 g, *Phragmites australis* (Cav.) Trin. ex Steud. [Poaceae; Phragmitis rhizoma] 30 g, *Coix lacryma-jobi var. ma-yuen* (Rom.Caill.) Stapf [Poaceae; Coicis semen] 30 g, *Platycodon grandiflorus* (Jacq.) A.DC. [Campanulaceae; Platycodonis radix] 9 g, *Pinellia ternata* (Thunb.) Makino [Araceae; Pinelliae rhizoma praeparatum cum zingibere et alumine] 12 g, *Allium chinense* G.Don [Amaryllidaceae; Allii macrostemonis bulbus] 12 g, *Amomum tsao-ko* Crevost and Lemarié [Zingiberaceae; Tsaoko fructus] 6 g, *Pogostemon cablin* (Blanco) Benth. [Lamiaceae; Pogostemonis herba] 10 g	N	N
Liu et al. (2020)	Lianhua Qingwen Capsules	Lianhua Qingwen Capsules (Shijiazhuang Yiling Pharmaceutical)	Pneumonia No.2 Formula: *Prunus armeniaca* L. [Rosaceae; Armeniacae semen amarum], *Ephedra sinica* Stapf [Ephedraceae; Ephedrae herba], *Ginkgo biloba* L. [Ginkgoaceae; Ginkgo semen], *Pheretima aspergillum* (E.Perrier) [Megascolecidae; Pheretima], *Descurainia sophia* (L.) Webb ex Prantl [Brassicaceae; Descurainiae semen], *Schisandra chinensis* (Turcz.) Baill. [Schisandraceae; Schisandrae chinensis fructus], *Pinellia ternata* (Thunb.) Makino [Araceae; Pinelliae rhizoma], *Glycyrrhiza uralensis* Fisch. ex DC. [Fabaceae; Glycyrrhizae radix et rhizoma], *Perilla frutescens* (L.) Britton [Lamiaceae; Perillae fructus], *Morus alba* L. [Moraceae; Mori cortex], *Tussilago farfara* L. [Asteraceae; Farfarae flos]	N	N
	Pneumonia No.2 Formula	Pneumonia No.2 Formula (-)			
Wang et al. (2021)	Qingfei Paidu Decoction	Hefei CR Sanjiu Medical and Pharmaceutical	*Glycyrrhiza uralensis* Fisch. ex DC. [Fabaceae; Glycyrrhizae radix et rhizoma praeparata cum melle] 6 g, *Ephedra sinica* Stapf [Ephedraceae; Ephedrae herba] 9 g, Gypsum Fibrosum 15–30 g, *Prunus armeniaca* L. [Rosaceae; Armeniacae semen amarum] 9 g, *Polyporus umbellatus* (Pers.) Fries [Polyporaceae; Polyporus] 9 g, *Cinnamomum cassia* (L.) J.Presl. [Lauraceae; Cinnamomi ramulus] 9 g, *Atractylodes macrocephala* Koidz. [Asteraceae; Atractylodis macrocephalae rhizoma] 9 g, *Alisma plantago-aquatica* L. [Alismataceae; Alismatis rhizoma] 9 g, *Bupleurum chinense* DC. [Apiaceae; Bupleuri radix] 16 g, *Poria cocos* (Schw.) Wolf [Polyporaceae; Poria] 15 g, *Scutellaria baicalensis* Georgi [Lamiaceae; Scutellariae radix] 6 g, *Iris domestica* (L.) Goldblatt and Mabb. [Iridaceae; Belamcandae rhizoma] 9 g, *Pinellia ternata* (Thunb.) Makino [Araceae; Pinelliae rhizoma praeparatum cum zingibere et alumine] 9 g, *Aster tataricus* L.f. [Asteraceae; Asteris radix et rhizoma] 9 g, *Zingiber officinale* Roscoe [Zingiberaceae; Zingiberis rhizoma recens] 9 g, *Pogostemon cablin* (Blanco) Benth. [Lamiaceae; Pogostemonis herba] 9 g, *Citrus × aurantium* L. [Rutaceae; Aurantii fructus immaturus] 6 g, *Citrus reticulata* Blanco [Rutaceae; Citri reticulatae pericarpium] 6 g, *Asarum sieboldii* Miq. [Aristolochiaceae; Asari radix et rhizoma] 6 g, *Dioscorea oppositifolia* L. [Dioscoreaceae; Dioscoreae rhizoma] 12 g, *Tussilago farfara* L. [Asteraceae; Farfarae flos] 9 g	N	N
Zhang et al. (2020c)	Modified Dayuan Formula	Sichuan Neo-Green Pharmaceutical	*Ephedra sinica* Stapf [Ephedraceae; Ephedrae herba praeparata cum melle] 10 g, *Prunus armeniaca* L. [Rosaceae; Armeniacae semen amarum] 15 g, Gypsum Fibrosum 20 g, *Trichosanthes kirilowii* Maxim. [Cucurbitaceae; Trichosanthis pericarpium] 20 g, *Rheum palmatum* L. [Polygonaceae; Rhei radix et rhizoma] 6 g, *Descurainia sophia* (L.) Webb ex Prantl [Brassicaceae; Descurainiae semen] 10 g, *Prunus persica* (L.) Batsch [Rosaceae; Persicae semen] 10 g, *Amomum tsao-ko* Crevost and Lemarié [Zingiberaceae; Tsaoko fructus] 6 g, *Areca catechu* L. [Arecaceae; Arecae semen] 10 g, *Atractylodes lancea* (Thunb.) DC. [Asteraceae; Atractylodis rhizoma] 10 g	N	N
Zhang et al. (2020d)	Jinyinhua Oral Liquid	Zhenao Honeysuckle Pharmaceutical	*Lonicera japonica* Thunb. [Caprifoliaceae; Lonicerae japonicae flos]	N	N
Luo et al. (2021)	Xuebijing Injection	Tianjin Chase Sun Pharmaceutical	*Carthamus tinctorius* L. [Asteraceae; Carthami flos], *Paeonia lactiflora* Pall. [Paeoniaceae; Paeoniae radix rubra], *Ligusticum striatum* DC. [Apiaceae; Chuanxiong rhizoma], *Salvia miltiorrhiza* Bunge [Lamiaceae; Salviae miltiorrhizae radix et rhizoma], *Angelica sinensis* (Oliv.) Diels [Apiaceae; Angelicae sinensis radix]	N	N
Xiong et al. (2020b)	Xuanfei Baidu decoction	-	*Ephedra sinica* Stapf [Ephedraceae; Ephedrae herba] 8 g, *Prunus armeniaca* L. [Rosaceae; Armeniacae semen amarum] 15 g, Gypsum Fibrosum 30 g, *Atractylodes lancea* (Thunb.) DC. [Asteraceae; Atractylodis rhizoma] 10 g, *Coix lacryma-jobi var. ma-yuen* (Rom.Caill.) Stapf [Poaceae; Coicis semen] 30 g, *Pogostemon cablin* (Blanco) Benth. [Lamiaceae; Pogostemonis herba] 15 g, *Reynoutria japonica* Houtt. [Polygonaceae; Polygoni cuspidati rhizoma et radix] 20 g, *Descurainia sophia* (L.) Webb ex Prantl [Brassicaceae; Descurainiae semen] 15 g, *Verbena officinalis* L. [Verbenaceae; Verbenae herba] 30 g, *Phragmites australis* (Cav.) Trin. ex Steud. [Poaceae; Phragmitis rhizoma] 30 g, *Artemisia annua* L. [Asteraceae; Artemisiae annuae herba] 25 g, *Citrus reticulata* Blanco [Rutaceae; Citri exocarpium rubrum] 20 g, *Glycyrrhiza uralensis* Fisch. ex DC. [Fabaceae; Glycyrrhizae radix et rhizoma] 10 g	N	N

### Risk of Bias of Selected Studies

**FIGURE 2:**
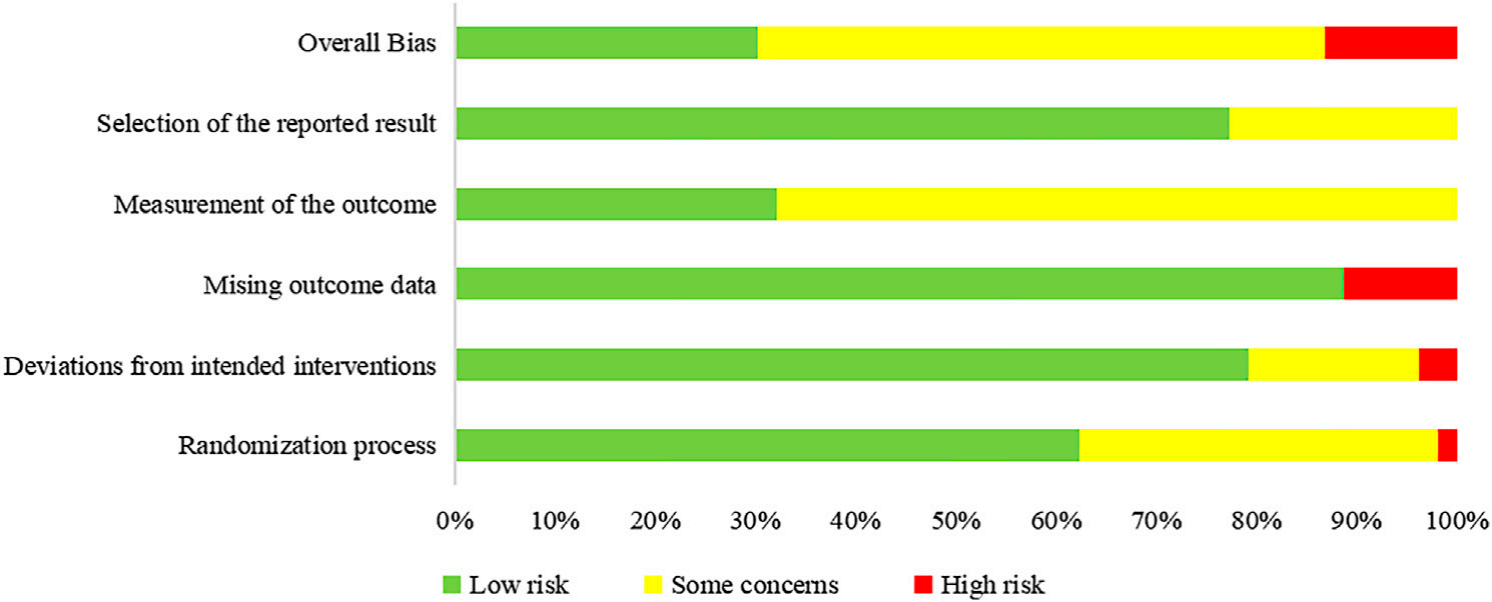
Summary of risk of bias.

We assessed the risk of bias of 58 outcomes in 25 RCTs: 21 outcomes in eight RCTs were assessed as “low risk” and were included in the meta-analysis, 30 as “some concerns,” and 7 as “'high risk.” Five trials did not report allocation sequence concealment, and 19 outcomes in these trials were assessed as “some concerns” in the randomization process (Fu et al., 2020; Li and Zhang, 2020; Qiu M. et al., 2020; Wen et al., 2020; Zhao et al., 2020a). One trial used patients’ hospitalization number to grouping and was assessed as “high risk” in the randomization process; the trial allocated odd-numbered patients to group A and allocated even-numbered patients to group B (Xiong W.-z. et al., 2020). Five trials (Qiu M. et al., 2020; Wang J.-b. et al., 2020; Xiong W.-z. et al., 2020; Zhang Y. L. et al., 2020; Duan et al., 2021) had deviations from the intended intervention and did not use an appropriate analysis (e.g., intention-to-treat [ITT] analysis); thus, nine outcomes in these trials were assessed as “some concerns” in deviations from intended intervention. One trial had imbalanced deviations between groups, and two outcomes were assessed as “high risk” (Sun H. M. et al., 2020). Four trials (Sun H. M. et al., 2020; Ye and CHAMPS Collaborative Group, 2020; Chen et al., 2021; Duan et al., 2021) did not report all the outcome data for nearly all participants randomized, and six outcomes in these trials were assessed as “high risk” in missing outcome data. Two objective outcomes (death and negativity of SARS-CoV-2 nucleic acid test), in which that assessment of the outcome cannot be influenced by knowledge of intervention received, were assessed as “low risk” in outcome measurement. Four studies (Fu et al., 2020; Hu et al., 2020; Wang J.-b. et al., 2020; Ye and CHAMPS Collaborative Group, 2020) were conducted in a blinded fashion to study allocation for outcome assessors, one trial (Luo et al., 2021) was a double-blinded RCT, and one trial (Yu et al., 2020) assessed the outcome with two independent assessors; eleven outcomes in these six trials were assessed as “low risk” in the outcome measurement. Eleven outcomes in nine trials (Ding et al., 2020; Lin et al., 2020; Qiu M. et al., 2020; Sun H. M. et al., 2020; Yu et al., 2020; Zhang C. T. et al., 2020; Zhao et al., 2020a; Chen et al., 2021; He and Zhang, 2021a) did not report measurements of outcomes and were assessed as “some concerns.” A summary of the risk of bias is shown in Figure 2.

### Clinical Cure

**FIGURE 3:**
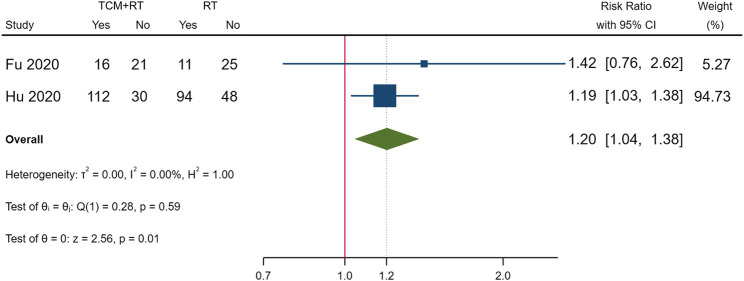
Forest plot of clinical cure.

Clinical cure was reported in nine RCTs; five trials used the TCM formula as the TCM intervention (Fu et al., 2020; Li and Zhang, 2020; Ye and CHAMPS Collaborative Group, 2020; Zhao et al., 2020a; Zheng et al., 2020), one trial used an oral Chinese patented drug (Hu et al., 2020), one trial used a Chinese medicine injection of Xuebijing (Chen et al., 2020), one trial used Chinese medicine extracts (Zhou W. M. et al., 2020), and one trial used the TCM formula and oral Chinese patented drugs (Liu W. et al., 2021). Three of the trials assessed as low risk of bias (Fu et al., 2020; Hu et al., 2020; Ye and CHAMPS Collaborative Group, 2020) and two trials that had a similar time point of outcome measurement were included in the meta-analysis (Fu et al., 2020; Hu et al., 2020). The result showed that TCM plus routine treatment could increase clinical cure better than routine treatment alone at 14–15 days (RR = 1.20, 95% CI [1.04, 1.38], *p* = 0.01) (Figure 3). An *I* ^2^ = 0% indicated that there was no heterogeneity between the two RCTs. A forest plot of the clinical cure is shown in Figure 3. Another study reported that no patients in either the TCM plus routine treatment group or routine treatment group were clinically cured at 7 days (Ye and CHAMPS Collaborative Group, 2020).

### Negativity of SARS-CoV-2 Nucleic Acid Test

**FIGURE 4:**
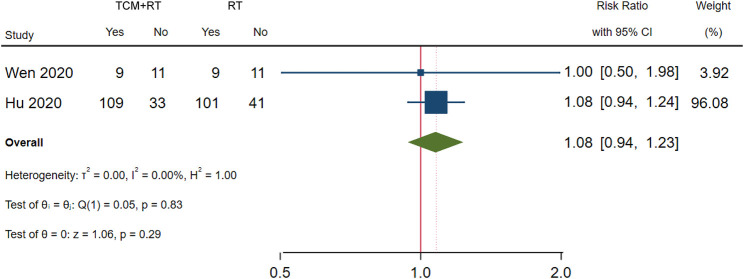
Forest plot of the negativity of the SARS-CoV-2 nucleic acid test.

The negativity status of the SARS-CoV-2 nucleic acid test was reported in 3 RCTs: one trial used the TCM formula as the TCM intervention (He and Zhang, 2021a), one trial used an oral Chinese patented drug of Lianhua Qingwen Capsules (Hu et al., 2020), and one trial used a Chinese medicine injection of Xuebijing (Wen et al., 2020). Two trials assessed as low risk of bias were included in the meta-analysis (Hu et al., 2020; Wen et al., 2020). The time point of the nucleic acid test was 7 days (Wen et al., 2020) and 14 days (Hu et al., 2020). No significant difference between TCM plus routine treatment and routine treatment alone was observed (RR = 1.08, 95% CI [0.94, 1.23], *p* = 0.29) (Figure 4). An *I* ^2^ = 0% indicated that there was no heterogeneity between the two RCTs. A forest plot of negativity of the SARS-CoV-2 nucleic acid test is shown in Figure 4. Another trial (Wang J.-b. et al., 2020) assessed as low risk of bias reported no significant difference in the time to the negativity of the nucleic acid test between the two groups (*p* = 0.263).

### Clinical Deterioration

**FIGURE 5:**
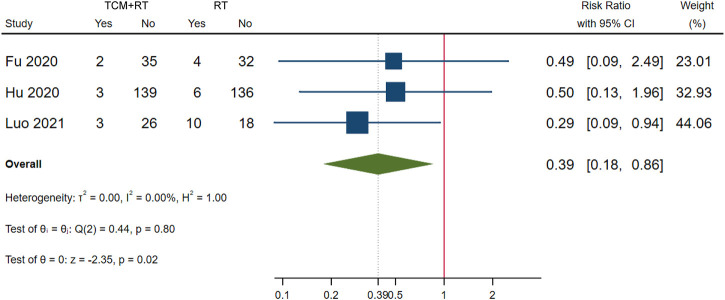
Forest plot of clinical deterioration.

Clinical deterioration was reported in 13 RCTs: four trials used the TCM formula as the TCM intervention (Fu et al., 2020; Lin et al., 2020; Qiu M. et al., 2020; Ye and CHAMPS Collaborative Group, 2020), six trials used oral Chinese patented drugs (Chen et al., 2020; Hu et al., 2020; Sun H. M. et al., 2020; Yu et al., 2020; Zhang Y. L. et al., 2020; Duan et al., 2021), two trials used a Chinese medicine injection of Xuebijing (Luo et al., 2021; Wen et al., 2020), and one trial used Chinese medicine extracts (Zhou W. M. et al., 2020). Four trials (Fu et al., 2020; Hu et al., 2020; Ye and CHAMPS Collaborative Group, 2020; Luo et al., 2021) were assessed as low risk of bias; three trials that had similar time points of outcome measurement were included in the meta-analysis (Fu et al., 2020; Hu et al., 2020; Luo et al., 2021). The meta-analysis showed that TCM plus routine treatment could prevent clinical deterioration better than routine treatment alone at 14–15 days (RR = 0.39, 95% CI [0.18, 0.86], *p* = 0.02) (Figure 5). An *I* ^2^ = 0% indicated that there was no heterogeneity between the three RCTs. A forest plot of clinical deterioration is shown in Figure 5. Another trial of low risk of bias (Ye and CHAMPS Collaborative Group, 2020) reported no difference in clinical deterioration rate between two groups of severe cases at 7 days (7.14 vs. 7.14%).

### Incidence of Unfavorable Clinical Events

**FIGURE 6:**
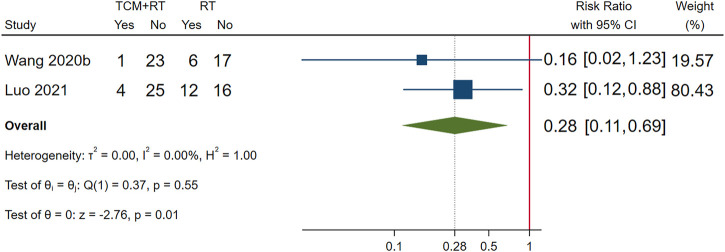
Forest plot of ARDS.

Incidence of ARDS was reported in 2 RCTs (Wang J.-b. et al., 2020; Luo et al., 2021): one trial used the TCM formula as the TCM intervention (Wang J.-b. et al., 2020) and one trial used Chinese medicine injection of Xuebijing (Luo et al., 2021). Both two trials were assessed as low risk of bias and were included in the meta-analysis. The result showed that TCM plus routine treatment could decrease the incidence of ARDS compared to routine treatment alone (RR = 0.28, 95% CI [0.11, 0.69], *p* = 0.01). An *I* ^2^ = 0% indicated that there was no significant heterogeneity between the two RCTs. A forest plot of chest image improvement is shown in Figure 6.

**FIGURE 7:**
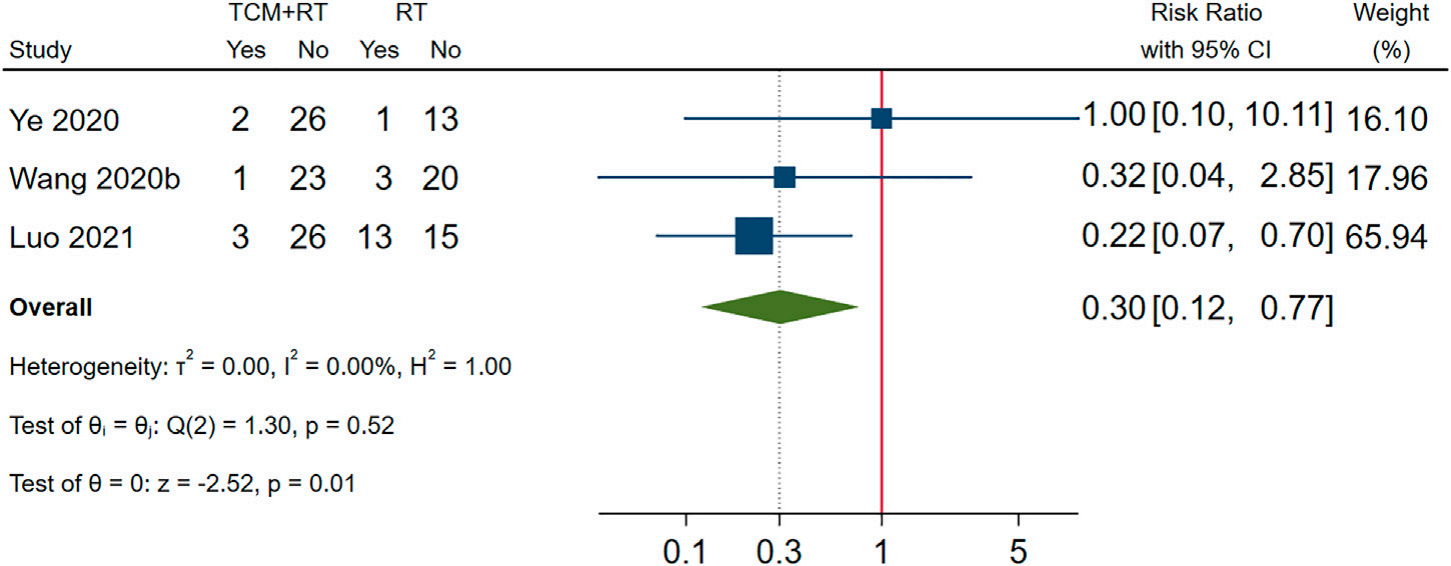
Forest plot of mechanical ventilation.

Incidence of mechanical ventilation was reported in 3 RCTs (Wang J.-b. et al., 2020; Ye and CHAMPS Collaborative Group, 2020; Luo et al., 2021): two trials used the TCM formula as the TCM intervention (Wang J.-b. et al., 2020) and one trial used Xuebijing injection (Luo et al., 2021). All three trials were assessed as low risk of bias and were included in the meta-analysis. The result showed that TCM plus routine treatment could decrease the incidence of mechanical ventilation compared to routine treatment alone (RR = 0.30, 95% CI [0.12, 0.77], *p* = 0.01). An *I* ^2^ = 0% indicated that there was no significant heterogeneity between the three RCTs. A forest plot of chest image improvement is shown in Figure 7.

The incidence of ICU admission was not reported as an outcome in the included trials, and thus meta-analysis was not conducted.

### Chest Image Improvement

**FIGURE 8:**
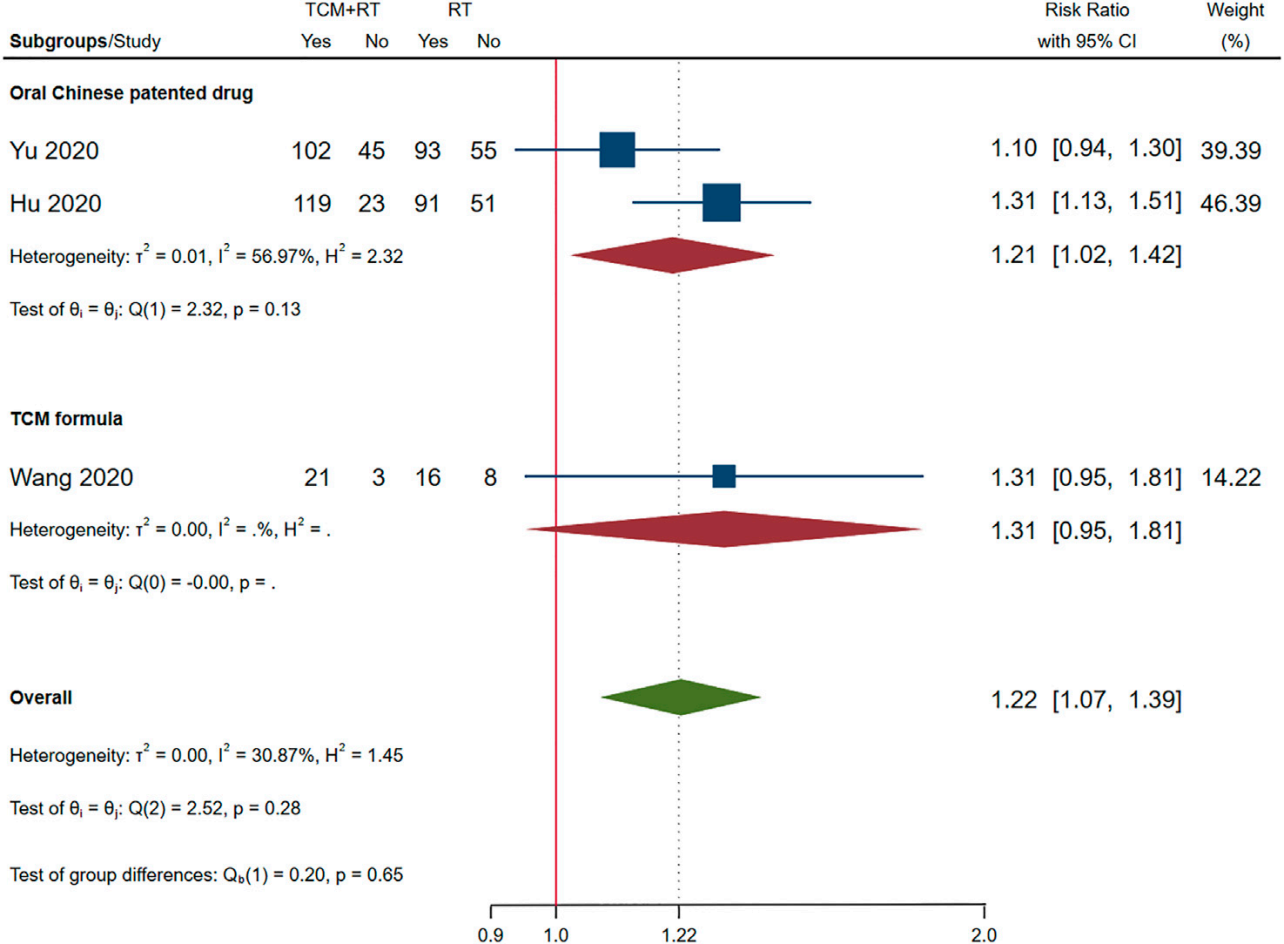
Forest plot of chest image improvements.

Chest image improvement was reported in 11 RCTs: eight trials used the TCM formula as the TCM intervention (Ding et al., 2020; Lin et al., 2020; Qiu M. et al., 2020; Wang J.-b. et al., 2020; Ye and CHAMPS Collaborative Group, 2020; Zhang C. T. et al., 2020; He and Zhang, 2021a; Wang et al., 2021) and three trials used oral Chinese patented drugs (Hu et al., 2020; Sun H. M. et al., 2020; Yu et al., 2020). Three trials assessed as low risk of bias were included in the meta-analysis (Hu et al., 2020; Wang J.-b. et al., 2020; Yu et al., 2020). The time point of chest image assessment was 7 days (Yu et al., 2020) and 14 days (Hu et al., 2020; Wang J.-b. et al., 2020). The result showed that TCM plus routine treatment was better than routine treatment alone (RR = 1.22, 95% CI [1.07, 1.39], *p* = 0.01). An *I* ^2^ = 30.87% indicated that there was no significant heterogeneity between the three RCTs. A forest plot of chest image improvement is shown in Figure 8. Subgroup analysis showed no significant difference between oral TCM patented drugs and the TCM formula (*p* = 0.65).

### Death

**FIGURE 9:**
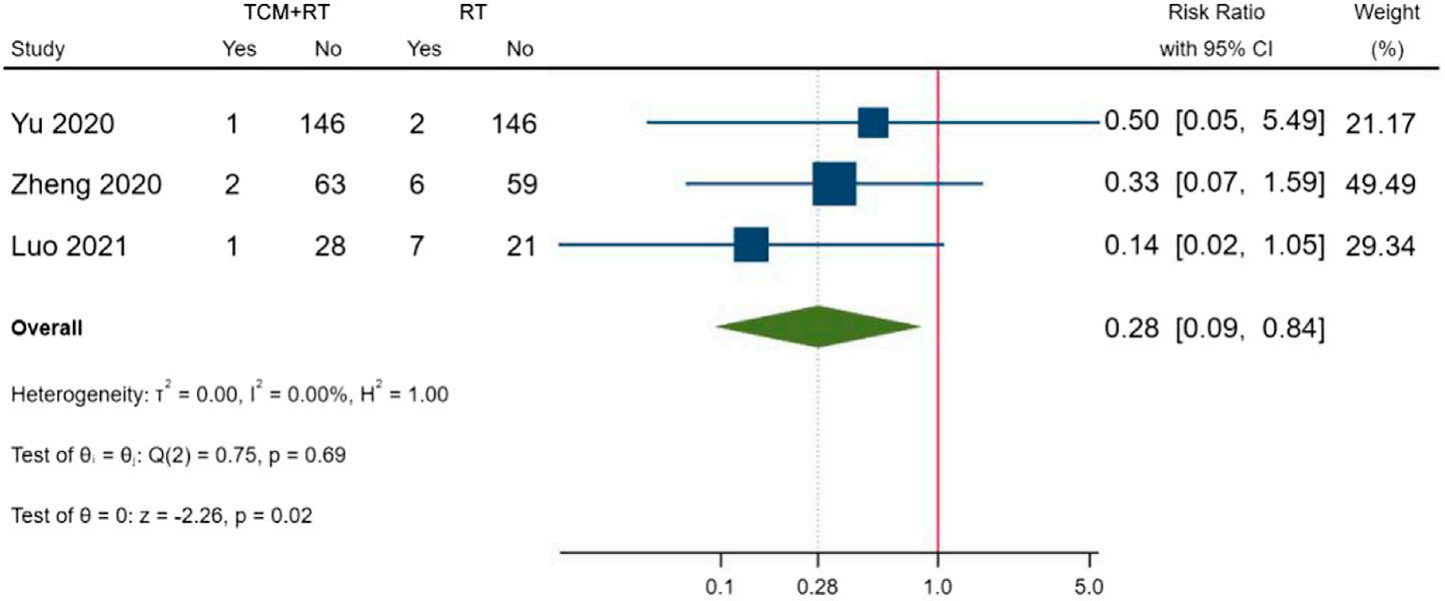
Forest plot of death.

Cases of death were reported in five RCTs: three trials used the TCM formula as the TCM intervention (Wang J.-b. et al., 2020; Ye and CHAMPS Collaborative Group, 2020; Zheng et al., 2020), one trial used an oral Chinese patented drug (Yu et al., 2020), and one trial used Xuebijing injection (Luo et al., 2021). Three trials assessed as low risk of bias were included in the meta-analysis (Yu et al., 2020; Zheng et al., 2020; Luo et al., 2021). The result showed that TCM plus routine treatment could decrease death compared to routine treatment alone (RR = 0.28, 95% CI [0.09, 0.84], *p* = 0.02) (Figure 7). An *I* ^2^ = 0 indicated no significant heterogeneity between the three RCTs. A forest plot of death is shown in Figure 9.

### Time to Fever Clearance

Time to fever clearance as the outcome was reported as an outcome in five trials: three trials used the TCM formula as the TCM intervention (Qiu M. et al., 2020; Wang J.-b. et al., 2020; Zhao et al., 2020a) and two trials used a Xuebijing injection (Chen et al., 2020; Luo et al., 2021). Only one trial (Luo et al., 2021) was assessed as low risk of bias; therefore, meta-analysis was not conducted for this outcome. The trial of low risk of bias (Luo et al., 2021) reported that the duration of fever in the Xuebijing injection group was shorter than that for the control group (5.54 ± 2.32 days vs. 7.34 ± 2.42 days, *p* = 0.018).

### Duration of Hospitalization

Duration of hospitalization was reported as an outcome in five trials, and all five trials used the TCM formula as the TCM intervention (Ai et al., 2020; Li and Zhang, 2020; Lin et al., 2020; Zhao et al., 2020a; Wang et al., 2021). All five trials were assessed as “some concerns”; thus, meta-analysis was not conducted. A significant reduction in the duration of hospitalization in TCM groups compared to routine treatment groups was reported in four trials (Ai et al., 2020; Li and Zhang, 2020; Lin et al., 2020; Wang et al., 2021), whereas another trial (Zhao et al., 2020a) reported no significant difference between the two groups.

### Adverse Events

Nineteen studies reported AEs as an outcome: seven trials (Chen et al., 2020; Ding et al., 2020; Hu et al., 2020; Wang J.-b. et al., 2020; Zhang Y. L. et al., 2020; Chen et al., 2021; Luo et al., 2021) reported that there was no obvious difference in the incidence of AEs between the TCM plus routine treatment group and routine group, five trials (Ai et al., 2020; Fu et al., 2020; Lin et al., 2020; Yu et al., 2020; Zhang C. T. et al., 2020) reported no treatment-related AEs in both groups, two trials (Wen et al., 2020; Xiong W.-z. et al., 2020) reported no TCM treatment-related AEs, three trials (Zhou W. M. et al., 2020; Liu W. et al., 2021; Wang et al., 2021) reported that TCM plus routine treatment could decrease the incidence of AEs more than routine treatment, and only one trial reported one serious AE in the routine treatment group and no serious AEs in the TCM plus routine treatment group (Wang J.-b. et al., 2020). One trial reported one allergic reaction in the TCM plus routine treatment group and no AEs in the routine treatment group (Li and Zhang, 2020). Another trial reported 27 AEs of diarrhea in the TCM plus routine treatment group, with eight patients stopping the medication on their own because of intolerance to diarrhea, and no AEs in the routine treatment group (Duan et al., 2021).

**FIGURE 10:**
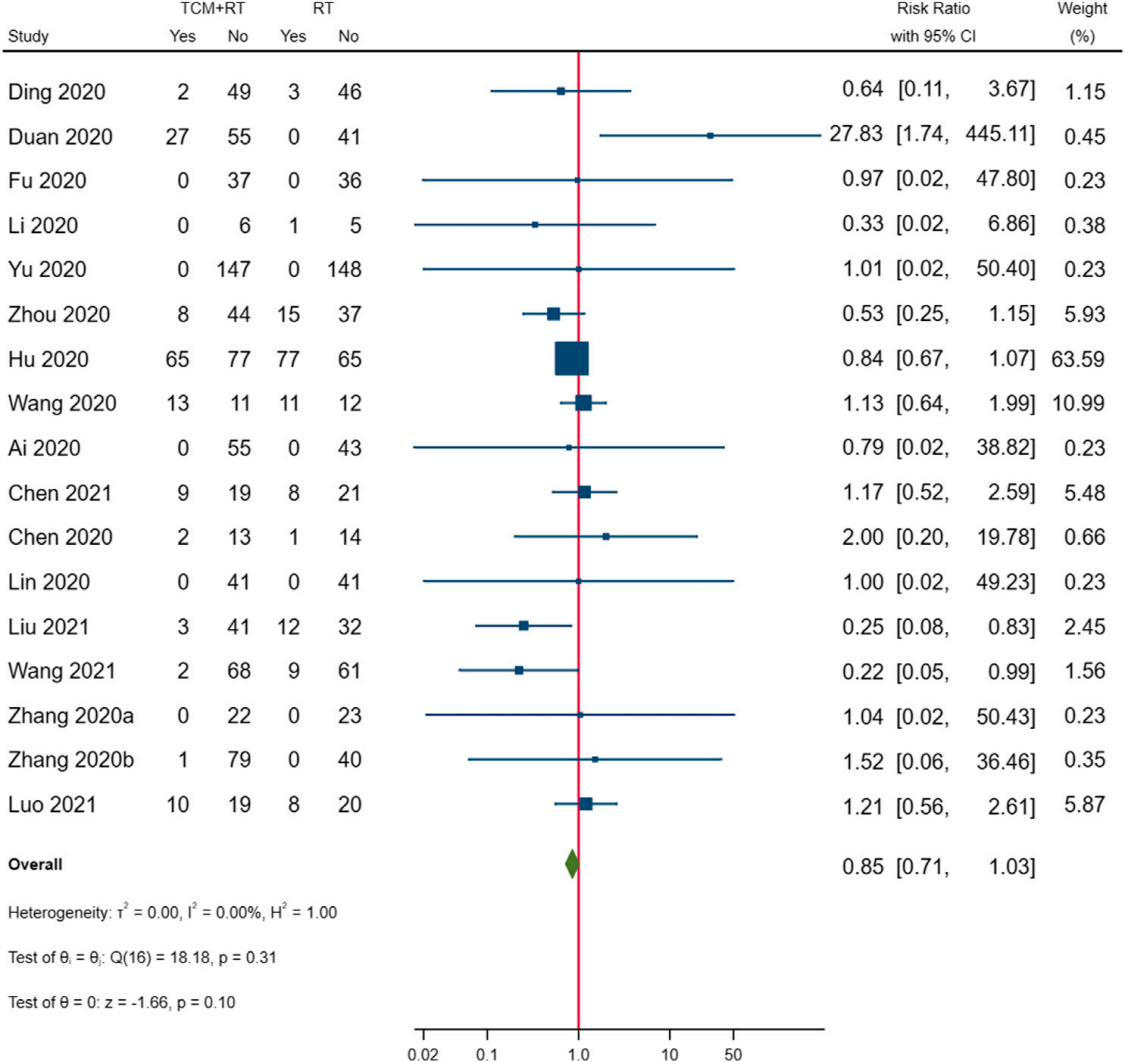
Forest plot of incidence of adverse events.

We synthesized the overall incidence of AEs reported in the 17 RCTs; two trials did not report AEs in the control groups and were not included in the meta-analysis (Wen et al., 2020; Xiong W.-z. et al., 2020). The result showed no significant differences in the overall incidence of AEs between the two groups (*p* = 0.10). The forest plot of incidence of AEs is shown in Figure 10.

### Subgroup Analysis

Because the number of studies included in the meta-analysis was small, subgroup analysis was only conducted for the chest image improvement outcome.

### Publication Bias

Owing to the limited number of studies included in the meta-analysis, a funnel plot and Egger’s test were not employed to assess the publication bias. Some publication bias was probably present since unpublished RCTs were not included in this SR.

### Quality of Evidence

The GRADE system was used to assess the quality of evidence. Evidence was assessed as moderate for clinical cure, clinical deterioration, ARDS, mechanical ventilation, death, and chest image improvement outcomes. For the negativity of the SARS-CoV-2 nucleic acid test, the quality was very low. A summary of findings is shown in Table 4.

**TABLE 4:** Summary of findings.

TCM plus routine treatment compared to standard treatment for COVID-19					
Patient or population: COVID-19 Setting: RCT Intervention: TCM plus routine treatment Comparison: Routine treatment					
Outcome № of participants (studies)	Relative effect (95% CI)	Anticipated absolute effects (95% CI)			Certainty
		Risk without TCM treatment	Risk with TCM treatment^a^	Risk Difference	
Clinical cure № of participants: 357 (2 RCTs)	**RR 1.20** (1.04 to 1.38)	59.0%	**70.8%** (61.3 to 81.4)	**11.8% more** (2.4 more to 22.4 more)	⊕⊕⊕○ MODERATE^b^
The negativity of SARS-CoV-2 nucleic acid test № of participants: 324 (2 RCTs)	**RR 1.08** (0.94 to 1.23)	67.9%	**73.3%** (63.8 to 83.5)	**5.4% more** (4.1 fewer to 15.6 more)	⊕○○○ VERY LOW^b^ ^,^ ^c^ ^,^ ^d^
Clinical deterioration № of participants: 414 (3 RCTs)	**RR 0.39** (0.18 to 0.86)	9.7%	**3.8%** (1.7 to 8.3)	**5.9% fewer** (8 fewer to 1.4 fewer)	⊕⊕⊕○ MODERATE^b^
ARDS № of participants: 104 (2 RCTs)	**RR 0.28** (0.11 to 0.69)	35.3%	**9.9%** (3.9 to 24.4)	**25.4% fewer** (31.4 fewer to 10.9 fewer)	⊕⊕⊕○ MODERATE^b^
Mechanical ventilation № of participants: 146 (3 RCTs)	**RR 0.30** (0.12 to 0.77)	26.2%	**7.8%** (3.1 to 20.1)	**18.3% fewer** (23 fewer to 6 fewer)	⊕⊕⊕○ MODERATE^b^
Chest image improvement № of participants: 627 (3 RCTs)	**RR 1.22** (1.07 to 1.39)	63.7%	**77.7%** (68.2 to 88.5)	**14.0% more** (4.5 more to 24.8 more)	⊕⊕⊕○ MODERATE^d^
Death № of participants: 482 (3 RCTs)	**RR 0.28** (0.09 to 0.84)	6.2%	**1.7%** (0.6 to 5.2)	**4.5% fewer** (5.7 fewer to 1 fewer)	⊕⊕⊕○ MODERATE^b^

a The risk in the intervention group (and its 95% confidence interval) is based on the assumed risk in the comparison group and the relative effect of the intervention (and its 95% CI). CI: Confidence interval; RR: Risk ratio

Explanations:

b Small sample size, the optimal information size criterion is not met.

c 95% CI overlaps no effect (RR of 1.0).

d The clinical heterogeneity between the trials exists, so we rate down for this outcome.

The bold was provided by GRADE to highlight the effect

### Summary of Evidence

With the RD calculated in Table 4 and the quality of evidence, we present our summary of evidence. The synthesized evidence showed moderate confidence of a benefit of 11.8% in clinical cure and 14.0% in chest image improvement and a reduction of 5.9% in clinical deterioration, 25.4% in ARDS, 18.3% in mechanical ventilation, and 4.5% in death with TCM treatment plus routine treatment compared to routine treatment alone in patients with COVID-19 (Figures 3, 5–9; Table 4). Low confidence of a benefit of 5.4% in the negativity of the SARS-CoV-2 nucleic acid test was also observed (Figure 4; Table 4). There were no significant differences in the overall incidence of AEs between the TCM plus routine treatment group and routine treatment group (Figure 10).

## Discussion

Our findings showed moderate confidence that TCM treatment of Toujie Quwen granules and Lianhua Qingwen Capsules plus routine treatment could promote a clinical cure, TCM treatment of Keguan-1 and Lianhua Qingwen Capsules plus routine treatment could promote chest image improvement, TCM treatment of Toujie Quwen granules, Lianhua Qingwen Capsules, and Xuebijing injection plus routine treatment could reduce clinical deterioration, TCM treatment of Keguan-1 and Xuebijing injection could reduce the development of ARDS, TCM treatment of Keguan-1, syndrome differentiation decoction, and Xuebijing injection could reduce the use of mechanical ventilation, and TCM treatment of syndrome differentiation decoction, Lianhua Qingwen Capsules, and Xuebijing injection plus routine treatment could reduce death compared to routine treatment alone in patients with COVID-19 (Figures 3, 5–9; Table 4). In addition, our findings showed that TCM treatment plus routine treatment may not promote the negativity of SARS-CoV-2 nucleic acid test compared to routine treatment alone (Figure 4; Table 4), and no significant differences were observed in the overall incidence of AEs between TCM plus routine treatment group and routine treatment group (Figure 10).

About 7.4–41.8% of COVID-19 patients developed ARDS (Huang et al., 2020; Rubin et al., 2020; Wu et al., 2020), and the mortality rate of COVID-19 patients with ARDS was 30.4–52.4% (Huang et al., 2020; Schlesinger et al., 2020; Wu et al., 2020). Pathoanatomy confirmed that COVID-19 is accompanied by a significant lymphocyte-predominant mononuclear inflammatory infiltrate (Tian et al., 2020). The nature of ARDS was an excessive and uncontrolled inflammatory response, forming a cytokine storm (Guan et al., 2020). TCM could promote immune balance and eliminate inflammation through cytokines-related pathways such as TLR and TNF (Peng et al., 2020). Ma Xing Shi Gan component inhibited the inflammatory response by interfering with TLR4/NF-κB/MAPK signaling pathway and reducing the release of inflammatory factors IL-1β, IL-6, and TNF-α (Yang R. et al., 2020). In addition, previous studies had found that a variety of phytochemical components contained in TCM such as flavonoids, alkaloids, terpenoids, polyphenols, and quinones can intervene in the occurrence, progression, and outcome of ALI/ARDS through a variety of mechanisms (He et al., 2021b). A double-blinded randomized controlled trial demonstrated that Xuebijing injection may suppress the cytokine storm and prevent the progression to ARDS in severe COVID-19 patients by regulating the secretion of pro-inflammatory cytokine IL-6, IL-8, and TNF-α (Luo et al., 2021). Another trial showed that Keguan-1 significantly improved the time to fever resolution and reduced the development of ARDS (Wang J.-b. et al., 2020). A retrospective single-center study found that TCM treatment of Shenhuang Granule significantly reduced the occurrence of ARDS (36.3 vs. 63.5%, *p* = 0.012) and the likelihood of receiving mechanical ventilation (66.7% vs. 72 84.7%, *p* = 0.028) and shortened the time from ICU admission to discharge (32 [20–73] days vs. 76 [63–79] days, *p* = 0.0074) (Feng et al., 2021). In addition, a retrospective study also found that in COVID-19, the mortality rate of cases that received TCM treatment was lower than that of cases that did not receive TCM treatment, whether in all cases or severe cases (6.2 vs. 35% for all cases; 22.1 vs. 77.7% for severe cases) (Shu et al., 2020). This synthesized evidence in this SR showed that the intervention of TCM treatment plus routine treatment could reduce the incidence of unfavorable clinical events of clinical deterioration, ARDS, mechanical ventilation, and death in patients with COVID-19. This evidence demonstrated that TCM treatment in the early stages may suppress the cytokine storm, prevent the progression to ARDS, decrease the use of mechanical ventilation, and eventually reduce the mortality of COVID-19 patients.

Our study had several strengths. We employed explicit eligibility criteria, conducted a comprehensive search of eight online databases, assessed eligibility and risk of bias critically, addressed important clinical efficacy-related outcomes, and assessed the quality of evidence using the GRADE system. Unlike 12 prior SRs (Ang et al., 2020; Jin L. et al., 2020; Luo et al., 2020; Pang et al., 2020; Qi et al., 2020; Sun C.-Y. et al., 2020; Wang S. X. et al., 2020; Xiong X. et al., 2020; Zeng et al., 2020; Zhang H. Y. et al., 2020; Zhang C. T. et al., 2020; Liu M. et al., 2021) that synthesized the data of both RCTs and observational studies in the same meta-analysis, this review excluded observational studies and updated the RCTs to summarize the latest evidence. We included ten newly published RCTs in this SR (Chen et al., 2020; Lin et al., 2020; Wen et al., 2020; Xiong W.-z. et al., 2020; Zhao et al., 2020a; Zheng et al., 2020; Chen et al., 2021; He and Zhang, 2021a; Luo et al., 2021; Wang et al., 2021) and a double-blinded RCT in the meta-analysis of the outcomes of clinical deterioration and death to synthesize new evidence (Luo et al., 2021). Furthermore, unlike other SRs that included both confirmed and suspected cases, this study excluded trials containing suspected cases. This study assessed the risk of bias of individual outcomes in the included RCTs with Risk of Bias Tool 2 but did not assess the risk of bias of individual studies. Unlike prior SRs that included both the low risk of bias studies and studies with “some concerns” or high risk of bias in a quantitative synthesis, this review only included outcomes with low risk of bias in the meta-analysis. We also assessed the quality of evidence critically using the GRADE system to a degree of confidence in the evidence.

There were several limitations in this SR. First, publication bias was probably present, as unpublished RCTs were not included in this systematic review. Second, only six of the 25 included studies were registered in the ChiCTR or in ClinicalTrials.gov, and selective reporting bias was not assessed rigorously. Third, only one trial was a double-blinded RCT, and only four trials used allocation concealment for outcome assessors. Finally, the evaluated treatments contained several different interventions and different courses of treatment in both TCM and routine treatments, thus leading to clinical heterogeneity among trials.

The time points of nucleic acid tests were baseline after randomization and at 14 days (Hu et al., 2020). In the early stages of the epidemic, nucleic acid tests were insufficient, which led to the negativity of the SARS-CoV-2 nucleic acid test being rarely reported as a primary outcome. It was reported that honeysuckle decoction inhibits SARS-CoV-2 replication and accelerates the negative conversion of infected patients (Zhou L. K. et al., 2020). However, we failed to conclude whether TCM accelerates negative conversion owing to limited evidence.

The risk of bias of included studies was critically evaluated, with only 30.2% (16/53) of outcomes being assessed as “low risk” in overall bias. The poor quality of clinical trials was a reason for the low quality of evidence in prior SRs (Ang et al., 2020; Xiong X. et al., 2020). Several reasons lead to the poor quality of included trials, but the leading cause was the absence of a blinded method, putting aside the huge number of patients and the shortage of human resources in the early stage of pandemic. The absence of a blinded method to outcome assessors caused poor performance in the measurement of outcome domain in RoB 2. Missing data and deviations from intended intervention may have also lead to poor quality. Finally, inappropriate analysis (e.g., per-protocol analysis) used to estimate the effect of the intervention may be another possible cause of the poor quality of the included trials.

Three of 25 included studies reported quality control of herbs or patented drugs (Hu et al., 2020; Wang J.-b. et al., 2020; Ye and CHAMPS Collaborative Group, 2020); the quality was in accordance with *The Pharmacopeia of People’s Republic of China.* Only one trial reported chemical analysis based on the analysis of the relative amounts of the standard compounds in components of Keguan-1 by high-performance liquid chromatography (HPLC) (Wang J.-b. et al., 2020). The standard compounds include chlorogenic acid, galuteolin, amygdalin, forsythoside A, forsythin, rutin, 3,5-dicaffeoylquinic acid, peimine, peiminine, and glyceryl trioleate (Wang J.-b. et al., 2020).

The results of this SR showed a moderate grade of confidence that TCM plus routine treatment promotes a clinical cure of COVID-19 patients compared to routine treatment alone. Our findings indicated a potential benefit of TCM integrated with western medicine in the treatment of COVID-19. The reason for the downgrade of the clinical cure is that the small sample size was below the optimal information size. We will update this study and the evidence when more rigorous RCTs with larger sample sizes are published in the future.

As the epidemic is mostly controlled in mainland China at this time, there are very few ongoing clinical trials of TCM on COVID-19 in the country. Some multi-center RCTs conducted in mainland China are in the process of publication. We searched ClinicalTrials.gov for clinical trials conducted overseas, and there was one ongoing trial in Singapore (Zhao, 2020b). Further RCTs of TCM and COVID-19 are still needed in countries where TCM treatment is legal and may be administered to patients.

## Conclusion

Synethized evidence of 21 outcomes in eight RCTs showed moderate certainty that TCM plus routine treatment could promote a clinical cure and chest image improvement compared to routine treatment alone while reducing clinical deterioration, development of ARDS, use of mechanical ventilation, and death in patients with COVID-19. TCM treatment plus routine treatment may not promote the negativity of the SARS-CoV-2 nucleic acid test compared to routine treatment alone. TCM treatment was found to be safe for patients with COVID-19.
