# Supplementary material for: Efficacy and Safety of Traditional Chinese Medicine in Coronavirus Disease 2019 (COVID-19): A Systematic Review and Meta-Analysis
Source: Front Pharmacol. 2021 Aug 6;12:609213. doi: 10.3389/fphar.2021.609213 (PMC8379002; doi:10.3389/fphar.2021.609213)
Supplement: Supplementary file 2 [file DataSheet2.doc]

**Search strategy will be used for Pubmed**

| Search | Query |
| --- | --- |
| #1 | Search (Coronavirus[MeSH Terms] OR COVID-19 [Supplementary Concept]) |
| #2 | Search ((((((((COVID-19[Title/Abstract]) OR 2019 novel coronavirus infection[Title/Abstract]) OR 2019-nCoV infection[Title/Abstract]) OR COVID-19 pandemic[Title/Abstract]) OR coronavirus disease-19[Title/Abstract]) OR 2019-nCoV disease[Title/Abstract]) OR COVID19[Title/Abstract]) OR 2019 novel coronavirus disease[Title/Abstract]) OR coronavirus disease 2019[Title/Abstract] |
| #3 | Search #1 OR #2 |
| #4 | Search (Medicine, Chinese Traditional[MeSH Terms]) OR Drugs, Chinese Herbal[MeSH Terms] |
| #5 | Search (((((((((((((((((((((((((Medicine, Chinese Traditional[Title/Abstract]) OR Traditional Chinese Medicine[Title/Abstract]) OR Traditional Medicine, Chinese[Title/Abstract]) OR Chinese Traditional Medicine[Title/Abstract]) OR Chinese Medicine, Traditional[Title/Abstract]) OR Drugs, Chinese Herbal[Title/Abstract]) OR Chinese Drugs, Plant[Title/Abstract]) OR Chinese Herbal Drugs[Title/Abstract]) OR Herbal Drugs, Chinese[Title/Abstract]) OR Plant Extracts, Chinese[Title/Abstract]) OR Chinese Plant Extracts[Title/Abstract]) OR Extracts, Chinese Plant[Title/Abstract]) OR Chinese medicine formula[Title/Abstract]) OR Chinese medicine decoction[Title/Abstract]) OR Qingfei Paidu Decoction[Title/Abstract]) OR Huashi Baidu Formula[Title/Abstract]) OR Xuanfei Baidu Formula[Title/Abstract]) OR Chinese patent medicine[Title/Abstract]) OR Chinese medicine Granule[Title/Abstract]) OR Chinese medicine Capsule[Title/Abstract]) OR Jinhua Qinggan Granule[Title/Abstract]) OR Lianhua Qingwen Capsule[Title/Abstract]) OR Lianhua Qingwen Granule[Title/Abstract]) OR Chinese medicine injection[Title/Abstract]) OR Xuebijing injection[Title/Abstract]) OR Xiyanping injection[Title/Abstract] |
| #6 | Search #4 OR #5 |
| #7 | Search #3 AND #6 |
|  | Filters: Randomized Controlled Trial |
